# Evidence for the effects of decommissioning man-made structures on marine ecosystems globally: a systematic map

**DOI:** 10.1186/s13750-022-00285-9

**Published:** 2022-11-01

**Authors:** Anaëlle J. Lemasson, Paul J. Somerfield, Michaela Schratzberger, Caroline Louise McNeill, Joana Nunes, Christine Pascoe, Stephen C. L. Watson, Murray S. A. Thompson, Elena Couce, Antony M. Knights

**Affiliations:** 1 School of Biological and Marine Sciences, University of Plymouth, Drake Circus, Plymouth, PL4 8AA UK; 2 PML –Plymouth Marine Laboratory, Prospect Place, The Hoe, Plymouth, PL1 3DH UK; 3 Cefas - Centre for Environment, Fisheries and Aquaculture Science, Lowestoft Laboratory, Pakefield Road, Lowestoft, NR33 0HT Suffolk UK

**Keywords:** Oil and gas, Offshore wind, Marine renewable energy, Artificial reefs, North sea, Synthesis, Repurposing, Reefing

## Abstract

**Background:**

Many marine man-made structures (MMS), such as oil and gas platforms or offshore wind turbines, are nearing their ‘end-of-life’ and require decommissioning. Limited understanding of MMS decommissioning effects currently restricts the consideration of alternative management possibilities, often leaving complete removal as the only option in certain parts of the world. This evidence-base describes the ecosystem effects of marine MMS whilst in place and following cessation of operations, with a view to informing decision-making related to their potential decommissioning.

**Method:**

The protocol used to create this map was published a priori. Systematic searches of published, literature in English were conducted using three bibliographic databases, ten specialist organisational websites or repositories, and one search engine, up to early 2021. A total of 15,697 unique articles were identified as potentially relevant to our research questions, of which 2,230 were screened at the full-text level. Of that subset, 860 articles met all pre-defined eligibility criteria. A further 119 articles were identified through “snowballing” of references from literature reviews. The final database consists of 979 articles. For each article included, metadata were extracted for key variables of interest and coded into a database.

**Review findings:**

The vast majority of eligible articles related to the presence of MMS (96.2%), while just 5.8% considered decommissioning. Overall, articles mainly considered artificial reefs (51.5% of all articles) but increasingly oil and gas (22%), shipwrecks (15.1%) and offshore wind (13.1%). Studies were distributed globally, but the majority focused on the United States, single countries within Europe, Australia, Brazil, China, and Israel; 25 studies spanned multiple countries. Consequently, the bulk of the studies focused on the North Atlantic (incl. Gulf of Mexico, North Sea, and Mediterranean Sea) and North Pacific Oceans. A further 12 studies had a global scope. Studies in majority reported on fish (53%) and invertebrates (41%), and were disproportionately focused on biological (81%) and ecological (48%) impacts. Physico-chemical (13%), habitat (7%), socio-cultural (7%), economic (4%) and functional (8%) outcomes have received less attention. The number of decommissioning studies has been increasing since ca. 2012 but remains noticeably low. Studies mostly focus on oil and gas infrastructures in the USA (Gulf of Mexico) and Northern Europe (North Sea), covering 9 different decommissioning options.

**Conclusions:**

This systematic map, the first of its kind, reveals a substantial body of peer-reviewed evidence relating to the presence of MMS in the sea and their impacts, but with considerable bias toward biological and ecological outcomes over abiotic and socio-economic outcomes. The map reveals extremely limited direct evidence of decommissioning effects, likely driven at least in part by international policy preventing consideration of a range of decommissioning options beyond complete removal. Despite evidence of MMS impacts continuing to grow exponentially since the early 1970s, this map reveals key gaps in evidence to support best practice in developing decommissioning options that consider environmental, social and economic effects. Relevant evidence is required to generate greater understanding in those areas and ensure decommissioning options deliver optimal ecosystem outcomes.

**Supplementary Information:**

The online version contains supplementary material available at 10.1186/s13750-022-00285-9.

## Background

The biological productivity and high economic resource value of the marine environment supports a wide range of human activities including shipping, aggregate extraction and fisheries [1]. For many activities, offshore man-made structures (MMS) are required. Since the discovery of exploitable oil and gas, marine constructions are estimated to cover > 32,000 km^2^ of our marine environment worldwide (1.5% of global Exclusive Economic Zones; [2]). Over time, this infrastructure reaches 'end of life' and requires decommissioning. This is defined as the fate of a structure following cessation of operations and/or activities for which it was originally deployed, and decommissioning may encompass all possible strategies and options from removal and disposal to repurposing and recycling.

Worldwide, much hard infrastructure is now at or nearing the end of its life. Globally it is estimated that > 7500 oil and gas (O&G) platforms in the waters of 53 countries will become obsolete over the next several decades, and most will require complete removal under current regulations [3] (but see OSPAR 98/3 amendment). Owing to their size, weight, and in some cases age, the removal of platforms and other MMS can be a complex engineering process, requiring some of the heaviest lifting operations ever attempted at sea and at great costs; the global cost of removal is estimated to exceed US$210 billion, with a substantial proportion of this cost provided to the industry through tax concessions [4]. However, while decommissioning of O&G is taking place, other structures (e.g. wrecks, cables and offshore windfarms [OWFs], but also further O&G installations) continue to be installed, often at a pace that exceeds the rate of decommissioning. For instance, in the UK, in 2020 just 10% of industry expenditure was on decommissioning; the remaining 90% was spent on exploration, development, and operations [5]. Much of this expansion of MMS is in response to climate change and inter-governmental pledges to reduce greenhouse gas emissions (e.g., UK national reduction target of 80% by 2050 compared to 1990 levels that is legally binding through the Climate Change Act [6]) and includes large increases in offshore wind generation capacity. Consequently, over the next 10 years, rapid expansion of offshore windfarms (OWFs) is planned [7], increasing their spatial footprint on the seabed. Offshore wind turbines, however, have a relatively short life-span (e.g. 10–15 years), such that > 1800 offshore wind turbines will require decommissioning between 2020 and 2030 [9], and this will involve environmental considerations similar to those for O&G platforms [4].

Despite acknowledgement that decommissioning will need to occur in both the short- and long-term, an assessment of the potential benefits, detriments, and trade-offs associated with different decommissioning strategies [9] is currently lacking. This is particularly true in regional seas (e.g., North Sea) where the political and legislative context is complex (see below reference to OSPAR), especially as local/national stakeholders with different perspectives may pursue different priorities and end-goals (see [10]). A strategy for decommissioning that benefits some stakeholders may be detrimental to others; e.g., a “Rigs-to-Reefs" strategy may create a de facto marine protected area, thereby contributing towards meeting conservation objectives, but may undermine fishers who consequently cannot physically deploy certain gears there [11]. One solution is to build consensus and use evidence-based decision-making and management of MMS and their decommissioning that: (1) is based on robust methodologies, (2) uses reliable and comprehensive evidence to provide the best possible advice to policy- and decision-makers, and (3) allows managers to make informed decisions about the trade-offs of alternate management actions [12, 13]. In places such as the North Sea, the legislation may need to be changed to enable the industry to adopt a wider range of options.

Although cessation of operations and decommissioning of MMS (e.g., O&G structures, but also some OWFs) is on-going, understanding of the environmental effects of different decommissioning strategies remains limited [4]. A narrow set of assessment criteria has also limited the evaluation of decommissioning effects [14] and restricted decommissioning options across seascapes. For instance, OSPAR Decision 98/3 on the Disposal of Disused Offshore Installations, which applies to the whole of the North East Atlantic Ocean, has in most cases restricted decommissioning options to complete removal (except specific exemptions, see [14]). Consequently, little consideration has been given to the potentially serious detriments of complete removal, or alternatively, potential benefits of alternative decommissioning options (such as repurposing them; see below). OSPAR Agreement 1999/13 (now 2012/3) Guidelines on Artificial Reefs in relation to Living Marine Resources, which were also influenced by the controversy underpinning Decision 98/3, has exacerbated this issue, stating that O&G infrastructure cannot easily be repurposed either *in-situ* or following relocation. Although OSPAR may appear restrictive, the legislation continues to be endorsed by the contracting parties and the EU commission.

There is a widespread acceptance that complete removal may not always be the most beneficial option [11, 15–17]. In some parts of the world, alternative decommissioning strategies have been undertaken, often with considerable success [16, 18]. For instance, some countries have allowed the relocation and/or alteration of O&G infrastructural components (generally jackets) to create artificial reefs (AR) (e.g., “Rigs-to-Reefs" programme in the USA, see [18, 19]) or have repurposed them by converting them to other uses (e.g., see review in [16] illustrating conversion to hotel and dive resorts, or their use in CO_2_ capture and storage (CCS)). In the UK, a number of sites are proposed for repurposing MMS reaching end-of-life, including those used for CCS [5]. Ultimately decommissioning strategies should be considered in light of the best current understanding and quantification of their effects on marine ecosystem structure, functioning and services.

There is a growing body of literature that advocates evidence-based environmental management and conservation [20–24], including in a marine context [25; but see 26], but a lack of systematic synthesis, summary, and/or accessibility despite concerted recent efforts (see [27–32]) has meant the evidence-base remains under-developed and under-used. In the context of MMS decommissioning, a large number of experts and stakeholders advocate an evidence-based multi-criteria approach to support decisions about decommissioning [33–38]. In the UK, industry operators must conduct Comparative Assessments (CA) of feasible options as part of their decommissioning proposals. For this assessment, they must address five main assessment criteria: safety, environmental, technical, societal, and economics [39, 40]. However, the evidence required to underpin such assessments as well as other multi-criteria approaches is not yet well established in either extent and/or availability, thereby hindering their application.

The cross-disciplinary nature of MMS decommissioning requires consideration of ecological, social, political, economic and technical aspects. Existing evidence appears to be dispersed across many literature sources and presented in a range of formats, some of which may be proprietary with access restricted to industry or government agencies. Additionally, and critically, there is very limited direct evidence (e.g., as pilot projects or experimental studies) for the effects of decommissioning, and the evidence used in key decision-making processes related to MMS (including CAs, but also licencing applications and decommissioning proposals) can lack peer-review or quantitative assessment [41]. Instead, the evidence is often found in industry-generated grey literature, often not peer-reviewed; as recently argued in the context of ocean sprawl [42], there may be conscious bias toward the use of certain literature which presents scenarios with a low(er) degree of environmental risk in order to pass licensing requirements and facilitate development. Irrespective of the reason, a comprehensive evidence base for the effects of decommissioning has been lacking such that revisions of conventions including OSPAR 98/3 or OSPAR 99/13 have been limited, hindering evidence-based decision-making and practice. To counter this limitation, several on-going projects are working to increase the evidence-base by generating new data and information, and/or collating existing evidence with a view to make it more readily accessible (such as Influence of Man-made Structures in the Ecosystem (INSITE) Programme phase 1 and 2, https://www.insitenorthsea.org). Although some studies are available which describe the effects of MMS and their decommissioning in the sea, no comprehensive collation of this type of evidence has been undertaken by systematic review or systematic map to date (but see [37, 43], and a response by [44], as well as [5, 45] on related topics).

Here, we present a systematic map (sensu [46]; defined further below) that focuses on published peer-reviewed research (primarily the academic literature—see “Methods” section) and documents the effects MMS have on marine ecosystems and ecological and socio-economic services associated with their presence and/or decommissioning. O&G infrastructure is included as well as OWFs, and other marine MMS that may be comparable to them in terms of their impacts, such as tidal energy installations, shipwrecks, artificial reefs, and carbon-capture and storage (CCS) installations. Here, the term “decommission” encompasses all possible strategies and options, from removal and disposal on shore to relocating or repurposing at sea. This evidence mapping exercise is part of the DREAMS project (*Decommissioning – Relative Effects of Alternative Management Strategies*, INSITE 2 programme [https://www.insitenorthsea.org]). DREAMS, as described in [47], aims to develop a system to show the relative effects of implementing different decommissioning strategies in the North Sea on a diverse range of ecosystem outcomes. As far as we are aware, while some studies have reviewed the literature around the effects of MMS while in place [5, 45], no studies have systematically synthesized the published literature around their decommissioning.

Systematic maps follow a rigorous, objective, and transparent evidence synthesis methodology, and collate and describe the captured evidence into a “catalogue” (see [46, 48] for further description of systematic maps). This collation and catalogue of all relevant and available published peer-reviewed research on the ecological and ecosystem service effects of MMS and their decommissioning in the marine environment makes the resultant evidence more available and accessible to all. This map can therefore play a key role in helping facilitate evidence-based decisions related to the management of marine MMS, including when assessing the “environmental” criterion of Comparative Assessments. It can also be used to identify critical research gaps that warrant future research.

Here, a systematic map methodology is better suited to the wide scope of our topic and our interest in the evidence distribution, rather than a systematic review methodology (see [48] for further discussion on the differences between systematic maps and systematic reviews). The evidence-base built in this map provides a foundation for multiple systematic reviews and meta-analyses (where appropriate) to answer more tightly focussed questions regarding the effects of MMS and their potential decommissioning options.

The results from the searches resulted in mapping the commercially published primary literature (excluding the grey literature); a resource not commonly used by policy- and decision-makers (see comments above), thereby allowing a determination of whether existing policy and decisions that may be based on industry-contracted grey literature provide similar or diametrically opposing evidence. By identifying and collating the available published evidence for the ecological and ecosystem service effects of marine MMS, this map: (i) informs subsequent systematic reviews and meta-analyses on related narrower topics; (ii) supports the parameterisation of numerical models for alternative scenario simulations; and (iii) facilitates evidence-based decision-making and the management of MMS in the marine environment.

### Stakeholder engagement and future work

This systematic map was developed in consultation with partner institutions involved in DREAMS: the University of Plymouth, Plymouth Marine Laboratory, the Centre for Environment, Fisheries and Aquaculture Science (Cefas), and Texas A&M University Corpus Christi. These may all be considered to be stakeholders. DREAMS is supported by the INSITE programme, an industry-science collaboration.

In addition, a small stakeholder group formed of expert representatives from academia, industry, and government agencies provided input into the evidence mapping project at the protocol stage [47].

## Objective of the review

A detailed description of the objectives of this systematic map are available in our protocol [47]. Briefly, the main objective was to identify and describe the evidence-base around the ecosystem and ecosystem service effects of the presence and decommissioning of MMS in the sea (i.e., during operations and after cessation of operations).

### Primary objectives (questions)

The primary questions for this systematic map were:

1. *What published evidence exists for the effects of marine man-made structures, while in place, on the marine ecosystem?*
2. *What published evidence exists for the effects of the decommissioning of marine man-made structures, on the marine ecosystem?*

The components of the primary questions were (further detailed below in “Eligibility criteria” section):

Population: Any components of the marine ecosystem (e.g.: ecosystems, assemblages or communities, populations or species, habitats, seabed or sediment, water column, humans/users of the sea). Geographical scope: global.

Exposure: Man-made structures (MMS) in the sea, during their operation, alterations and any options for their decommissioning. MMS included: oil and gas (O&G) installations, offshore wind farms (OWF), marine renewable energy installations (MREI – such as tidal wave devices) shipwrecks, artificial reefs (AR), carbon capture and storage (CCS) facilities, and any other relevant and similar MMS (such as offshore research platforms).

Comparator: Over time (After Only; Before/After), over space (Control/Impact; inside/outside; control or reference site), over time and space (Before-After-Control-Impact), no comparator and correlative studies.

Outcome: Change in any aspects of the marine ecosystems (i.e. all possible outcomes).

Further information regarding the nature and scope of the primary question components (e.g. the subject population, exposure, comparators, and outcome measure) can be found in our protocol and below under “Eligibility criteria”.

### Secondary objectives

The secondary questions for this systematic map were:

3. *Which decommissioning options have been ‘well’ studied (knowledge clusters), and which ones are lacking published evidence (knowledge gaps)?*
4. *What is the distribution and abundance of studies between outcomes/metrics, populations (*sensu* “PECO”), geographical locations, structure types/age, and years?*

## Methods

This map follows our protocol previously published in this journal [47] and followed the Collaboration for Environmental Evidence Guidelines and Standards for Evidence Synthesis [48]. The mapping methods conform to the RepOrting standards for Systematic Evidence Syntheses (ROSES) for systematic maps [49] (Additional file 1).

### Deviations from the protocol

The methods used to conduct this systematic map followed those described in the published protocol [47] with some small deviations described below. Briefly, they include: changes to the number of organisational websites searched and their search methodology; extending the population to “other similar marine man-made structures”; extending the study designs to qualitative social studies (where relevant to our primary question); and addition of categories used during coding (e.g., presence or mention of non-native species).

### Search for articles

#### Search terms and strings

Because our primary objective was broad and our aim was to collate the evidence about any effects of MMS on the marine environment, no specific Outcome terms were included in the search string. This allowed the search string to retrieve studies related to all possible outcomes (see our protocol [47] for further explanation).

The full search string (formatted for Web of Science) was built by combining the following strings for each group:

*Marine and offshore qualifying terms*

TS = (marine OR offshore OR pelagic OR benthic OR ocean* OR sea OR shelf OR shelves OR coast*)

AND

*Population terms*

TS = (ecosystem$ OR habitat$ OR seabed$ OR sediment$ OR “ecological system*” OR “water column” OR benthos OR environment* OR species OR assemblage$ OR communit* OR population$ OR fisher* OR service$ OR human$ OR people).

AND

*Exposure terms*

TS = (plac* OR install* OR deploy* OR decommission* OR manag* OR reefing OR toppling OR topping OR repurpose* OR relocat* OR alter* OR salvag* OR remov*) AND.

*Man-made structure terms*

TS = (“man-made structure$” OR “offshore structure$” OR “artificial structure$” OR “oil and gas” OR “oil and gaz” OR “oil & gas” OR “oil & gaz” OR “oil rig$” OR “petroleum installation*” OR “windfarm$” OR “wind farm$” OR "wind turbine*" OR MREI OR "wave farm*" OR "tidal energy" OR "tidal stream*" OR “artificial reef$” OR wreck$ OR “shipwreck$” OR CCS OR “carbon capture” OR “carbon storage”).

Search strings formatted for Scopus, AFSA, and Google Scholar are available in Additional file 2.

### Sources of literature to be searched and limitations

#### Databases, search engines, and organisational websites

In total, three bibliographic databases (Web of Science Core Collection, Scopus, and Aquatic Sciences and Fisheries Abstracts), one search engine (Google Scholar), and 10 institutional and organisational websites were searched between November 2020 and March 2021 using the University of Plymouth subscription (Table 1, see also Additional file 2). Searches were undertaken in bibliographic databases until February 1^st^ 2021. In addition, institutional and organizational websites were searched between February ee (with the exception of the Cefas Data Hub and the INSITE website and database which were searched in November 2020). The search engine Google Scholar was queried in November 2020 and the first 200 returns selected to supplement our searches. Searches were undertaken in English only. Although we stated in our protocol that searches would also be undertaken in French (language spoken by the review team and relevant to the North-Sea context) if time allowed; this was forgone due to insufficient time.

**Table 1:** List of literature sources that were systematically searched for relevant studies

Literature source
Web of Science Core Collection^ab^
Scopus^b^
Databases within the Aquatic Sciences and Fisheries Abstracts publisher platform ProQuest^b^
Collaboration for Environmental Evidence library^c^
INSITE database and website^c^
Environmental Studies Program Information System (ESPIS) repository^d^
Centre for Environment, Fisheries & Aquaculture Science (Cefas Publication Hub)^d^
Wageningen University & Research repository^d^
Royal Belgian Institute of Natural Science repository^d^
Alfred Wegener Institute repository^d^
Royal Netherlands Institute of Sea Research (NIOZ) repository^d^
Collaborative Offshore Wind Research Into The Environment database (COWRIE)^c^
Nature-based Solutions Initiative evidence platform^c^

^a^WoS was searched using the University of Plymouth subscription, citation indices are listed in Additional file 2

^b^Sources searched using the agreed search string (or a variant thereof)

^c^Sources where our search string could not be entered and a manual hand-search was performed

^d^Sources where keywords searches were used

Where searches could not be performed using the agreed search string or a modified syntax of the search string—for instance on some organisational websites—either manual systematic hand-searches or keyword searches were performed (see Table 1 for details). Keyword searches were not initially planned and are a deviation from our protocol; this is because some websites did not allow access to the full publication list and could only be browsed by entering search terms. Keyword searches were performed using the following terms: “oil and gas”, “oil and gaz”, “oil & gas”, “oil & gaz”, “tidal energy”, “platform”, “offshore”, “man-made structure”, “artificial reef”, “wind farm”, “windfarm”, “shipwreck”, “ship wreck”, and “wreck”.

**Fig. 1:**
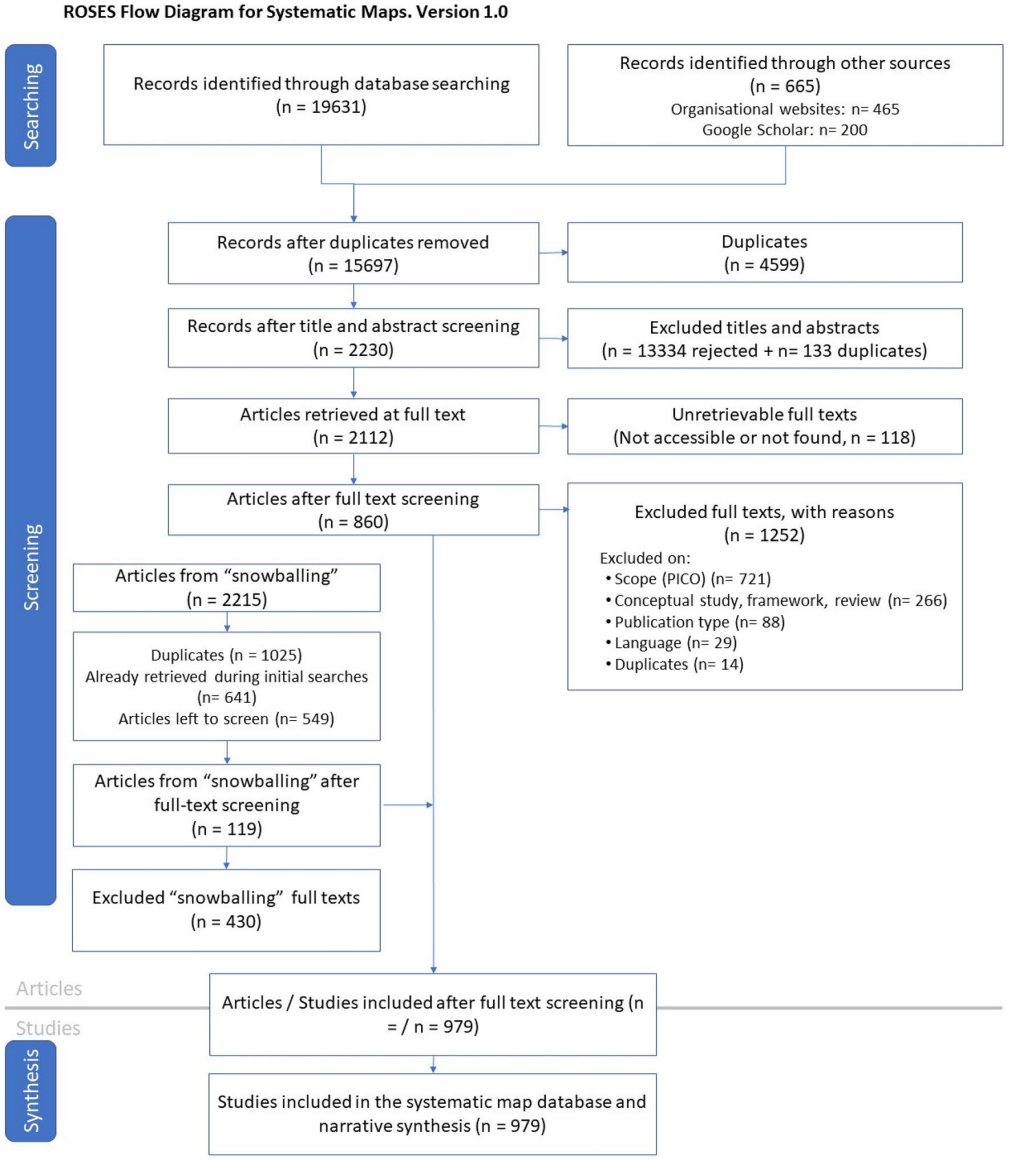
ROSES flow diagram detailing the systematic mapping process

Articles that passed the first stage of screening (at title and abstract level) were included (see Fig. 1: ROSES flow diagram and Additional file 3).

Due to time constraints, we were unable to search all the literature sources identified in our protocol. These had been split into “priority sources” and “secondary sources” (see protocol), and the following sources identified as “secondary” were not searched: Department for Environment, Food and Rural Affairs*,* Joint Nature Conservation Committee, Marine Scotland, Natural Resources Wales (Marine and coastal evidence reports), English Nature Access to Evidence, Environment Agency, Marine Management Organisation, International Council for the Exploration of the Sea, Helsinki Convention (HELCOM), Oslo-Paris Commission (OSPAR), BSH (Federal Maritime and Hydrographic Agency), Offshore Petroleum Regulator for Environment and Decommissioning (OPRED). Applied Ecology Resources was not searched because the website was not live yet at the time of searches.

As described in our protocol, the comprehensiveness of the search was assessed using a list of 25 benchmark articles compiled by the members of the review team, of which 24 (96%) were retrieved using our search strategy (see Additional file 2).

#### Other searches (snowballing)

Where a systematic review or a meta-analysis was found and passed the screening process, all studies reviewed within it were included, as well as the meta-analysis. The systematic review itself was included only if it contained new primary data. Where a verbose (non-systematic) review was found, all relevant studies referenced within it were subjected to the screening process and included if they passed it, but the review itself was not, unless it also provided new or collective data. Other articles that passed the screening process and were included in the systematic map were not scanned for relevant citations due to time restrictions and the low likelihood of finding new relevant studies.

### Assembling a library of search results

As detailed in the protocol, articles retrieved following our searches were entered into a single library in the reference manager Zotero. Duplicate entries were removed prior to the screening process in Microsoft Excel.

## Article screening and study eligibility criteria

### Screening process

Articles identified during initial searches in databases, search engines, and organisational websites were assessed for inclusion according to a 2-step hierarchical assessment of relevance: (1) at title and abstract level, and (2) at full text level. At each stage, an article was included or excluded from the map based on the agreed eligibility criteria (see protocol and below for details). Where the relevance of an article was unclear at stage 1, it was included and assessed at stage 2, to err on the side of inclusion. We recorded the number of articles included/excluded at each stage (see Fig. 1). Records were kept of all the articles excluded at stage 2 (full text), together with the reasons for their exclusion (see Additional file 3). Similarly, records were kept of articles that could not be located or accessed. Where an article could not be located or accessed in full using the University of Plymouth subscription, we attempted to contact the authors with a request for the full text. Articles identified through the “snowballing” exercise were assessed separately, with the same reviewer undertaking step (1) and (2) concurrently. Due to time constraints, where an article identified through snowballing could not be located or accessed, we did not attempt to contact the authors. All these records are available in Additional files 3 and 4.

A team of nine independent reviewers participated in the first stage of the screening process, whilst a team of six participated in the second stage and in the snowballing exercise. Consistency checking was performed at each stage on a subset of articles by all reviewers involved. For stage 1, an initial random subset of 100 articles was independently screened by all reviewers for inclusion/exclusion, returning a level of agreement of 55%, which was below the acceptable level. Following additional training and discussion around our PECO criteria in order to resolve differences and improve consistency in eligibility decisions, a second subset of 50 random articles was screened, returning an acceptable level of agreement of 76%. For stage 2, a random subset of 30 articles was independently screened by all reviewers for inclusion/exclusion, returning an acceptable level of agreement of 65% (for consistency related to data coding, see “Data coding strategy” below). Agreement levels were assessed using Cohen’s Kappa test [50].

In the instance that a retrieved article was authored or co-authored by one or more members of the review team, the article was referred to another reviewer for assessment.

### Eligibility criteria

Our eligibility criteria were described in detail in our protocol [47]. Where deviations have occurred, we specify them here.

#### Eligible population/subject

Unchanged from our protocol. All relevant marine components, including ecosystems, populations, species, communities, assemblages, as well as the water column, habitats, sediments and the seabed were considered. Additionally, we considered humans as a population, for instance for evidence related to ecosystem services. The geographical scope considered was global (i.e.: evidence from all marine environments globally) to allow us to draw comparisons between geographical locations. It should be noted that we only considered fully marine contexts, and did not include studies undertaken in freshwater or estuarine environments.

#### Eligible exposures

We included all MMS listed in our protocol. These included: O&G structures, OWF, tidal energy installations and other marine renewable energy installations (MREI), shipwrecks, artificial reefs (ARs), and CCS. In the rare instances where a study dealt with a MMS that was deemed relevant and associated by the study authors to one of the eligible MMS types, we also included it and coded it under “Other similar MMS” (e.g., research platform, sea fort). Please note that we considered “artificial reefs” only those structures deployed intentionally as ARs, and not structures originally intended for other purposes but deemed by the study authors to constitute ARs (e.g. breakwaters, jetties, seawalls, etc.).

#### Eligible comparators

We included studies that used the eligible comparators listed in our protocol. Those included: temporal comparators (before/after, time series), spatial comparators (between different structures or sites, near/inside vs far/outside a structure or site, as well as depth comparisons), as well as procedural controls and reference sites (Control/Impact). For instance, a study could compare one specific intervention/exposure against another, such as comparing the outcomes of different decommissioning strategies. We also considered studies that used a combination of temporal and spatial comparators (Before-After-Control-Impact designs). In addition, we also included studies that did not include a strict comparator and were of a more correlative nature, where relevant and containing useful information.

#### Eligible outcomes/metrics

As described above, we considered all possible outcomes, by including studies that assessed any ecosystem effects on any components of the marine environment. Our protocol listed some of the anticipated relevant outcomes/metrics: ecological and biological effects (such as ecosystem function, ecosystem structure, community/assemblage composition, diversity, species presence/absence, abundance, biomass, habitat type and quality etc.); physical and geochemical effects (such as grain size, sediment type, flow); effects on connectivity (e.g. propagule and larval dispersal; population connectivity); as well as ecosystem service effects and social and economic effects where relevant and directly related to an ecosystem service outcome (such as fisheries displacement). Additional ones were added *ad-hoc* during the coding stage if not already listed.

#### Eligible study designs

In our protocol, we stated that we would include evidence from either (1) studies that have experimentally tested, measured or assessed effects, (2) observational studies that have recorded or quantified effects or relevant outcomes, (3) systematic reviews and meta-analyses. In addition to the eligible study designs listed in our protocol, we also included qualitative social studies, including those which dealt with public or stakeholder’s perceptions of MMS and their effects on the marine environment. We did not include modelling studies, purely theoretical or conceptual studies, nor did we include verbose reviews, unless they provided or were based upon new empirical quantified effects.

### Study validity assessment

For this systematic map, the validity of each piece of evidence was neither assessed nor weighed. However, information regarding the design of each study was coded and so can be considered by the users of the map when interpreting the evidence. The validity of relevant studies will be assessed during the production of the planned systematic reviews and meta-analyses.

### Data coding strategy

Meta-data (information describing each study) were extracted and coded for all articles retained in the final database (N = 979), following a standardised coding framework. Note that a single article can describe more than one study, but here we extracted the meta-data per article, with each article given a unique identifier. We coded the following: (1) PECO components (as described above in “Eligibility criteria” section); these included population (at three hierarchical levels), exposure (at one or two levels), study design/comparator (at two levels) and outcome (at two levels). (2) Additional study information, such as study year and duration, study location (at three geographical levels), structure identity and structure type (at two or more levels), structure depth (or height if applicable), and water temperature at the study site. (3) In addition to these categories stated in our protocol, we also coded whether the article mentioned non-native or invasive species, whether it mentioned commercial or recreational species, and we also provided a brief account of the article topic. (4) We coded bibliographic information related to the article (article reference; article year of publication; journal/report name). Finally, (5) we coded reviewer information (which team member extracted the data). When information was missing or unclear, we clearly stated it by coding the associated fields with the term “unspecified” or “unclear”, respectively.

To ensure the consistency and accuracy of data extraction, and validity of data coding, the same set of articles as the one used for the consistency assessment of screen 2 (see “Screening process” section) was used to assess consistency in meta-data extraction and coding. Out of the 30 articles, 24 passed screen 2, and their meta-data were extracted and coded by all members of the review team. Agreement levels were assessed for each of the following categories: Final inclusion; Population level 2; Exposure level 1; Study design level 1; Structure type; Geographical location level 1; and Country of study. This returned on average a level of agreement of 79% (± 15). Agreement levels were assessed using Cohen’s Kappa test [49]. Disagreements were again discussed in order to improve consistency in data coding.

### Data mapping method

The evidence base identified is presented as a coded queryable Microsoft Excel database (Additional file 4). It was explored and summarised using both Excel and the package dplyr [51] in R. Data were plotted using a combination of ggplot [52], tmap [53], and Excel. Geographical maps were initially created in R (code provided in Additional file 5, based on the data in the Excel sheet in Additional file 6), then further modified for aesthetic purposes in Adobe Illustrator. Data were summarised by MMS exposure type, geographic location, structure type, study design, population and outcome measures, or by year to show knowledge gaps and knowledge clusters using various figure formats. For instance, geographic data are presented using geographical maps of countries and sea areas to illustrate the number of articles associated with them, and heatmap matrices are used to show the distribution and frequency of evidence grouped by categories.

Based on these results, we make recommendations on priorities for future research related to the management and decommissioning of marine MMS. As mentioned above, these results will also inform subsequent systematic reviews and meta-analyses on related narrower questions pertaining to the effects of marine MMS and their decommissioning. Examples of a priori planned systematic review questions include “*What are the effects of different types of marine MMS on emergent epifaunal communities”, and “What are the effects of repurposing an MMS into an AR on marine biodiversity/fish population/species abundance/composition*”.

## Review findings

In total, 20,296 records were retrieved from searches across bibliographic databases (19,631), organisational websites (465), and Google Scholar (200); these held 4,599 duplicates (Fig. 1). Of the 15,697 records left, most articles were excluded at title and abstract screening due to not being relevant (13,334) or being duplicates (133), leaving 2,230 unique records for full-text screening. The full texts for a small number of articles (118; 5%) were unretrievable, leaving 2,112 articles for full-text screening. Of these, 1,252 articles were excluded from the map based on either scope (n = 721; PECO), study type (n = 266; e.g. conceptual study, review, framework), publication type (n = 88; e.g. report, book, conference paper), language (n = 29), lack of data (n = 134) or duplication (n = 14). This left 860 articles.

During the screening process, an additional 2,215 articles were identified through 'snowballing'. Of those, 1,025 were duplicates and 641 had already been retrieved during the initial search, leaving 549 articles for assessment. Screening indicated that 119 (22%) contained relevant information based on the search criteria, and the remaining 430 (78%) were excluded.

In total, 979 articles are included in the systematic map after full-text screening of articles identified following the initial search and secondary snowballing process.

Due to the particular context of the marine environment, most articles retrieved described studies relating to multiple Populations, Outcomes, and even sometimes study designs (Comparators). A great number of studies could thus be extracted from a single article (e.g.: if an article describes the effects of the presence of an artificial reef and a shipwreck on fish and invertebrates, this could be interpreted as up to four different studies). For ease of interpretation and discussion, we decided in this report to refer to the unique articles as studies, hence we consider our systematic map to consist of 979 studies. Hereafter, we use the terms ‘study’ and ‘article’ interchangeably.

Mapping the quantity of studies relevant to the primary questions.

### Distribution of evidence by Exposure type

The majority of articles (942) focused on the 'Presence of MMS' (96.2% of the 979 unique articles), while just 57 (5.8%) focused on 'Decommissioning of MMS'. We only found 6 articles (0.6%) describing studies with the exposure type 'Alteration of MMS (not part of decommissioning)' (Table 2). Further results relating specifically to decommissioning studies are described and discussed below (see ‘Distribution of evidence related to decommissioning’ section). Hereafter, results presented relate to articles for all exposure types combined.

**Table 2:** Count of articles by exposure types

Exposure type	Count of articles	Percentage of unique articles*
Presence of MMS	942	96.22%
Decommissioning of MMS	57	5.82%
Alteration of MMS	6	0.61%
Grand Total*	1005	

*Grand total may be greater than the number of unique articles (N = 979), and percentages may add up to more than 100%, as some articles contains studies spanning multiple exposure types

### Distribution of evidence by MMS types

**Fig. 2:**
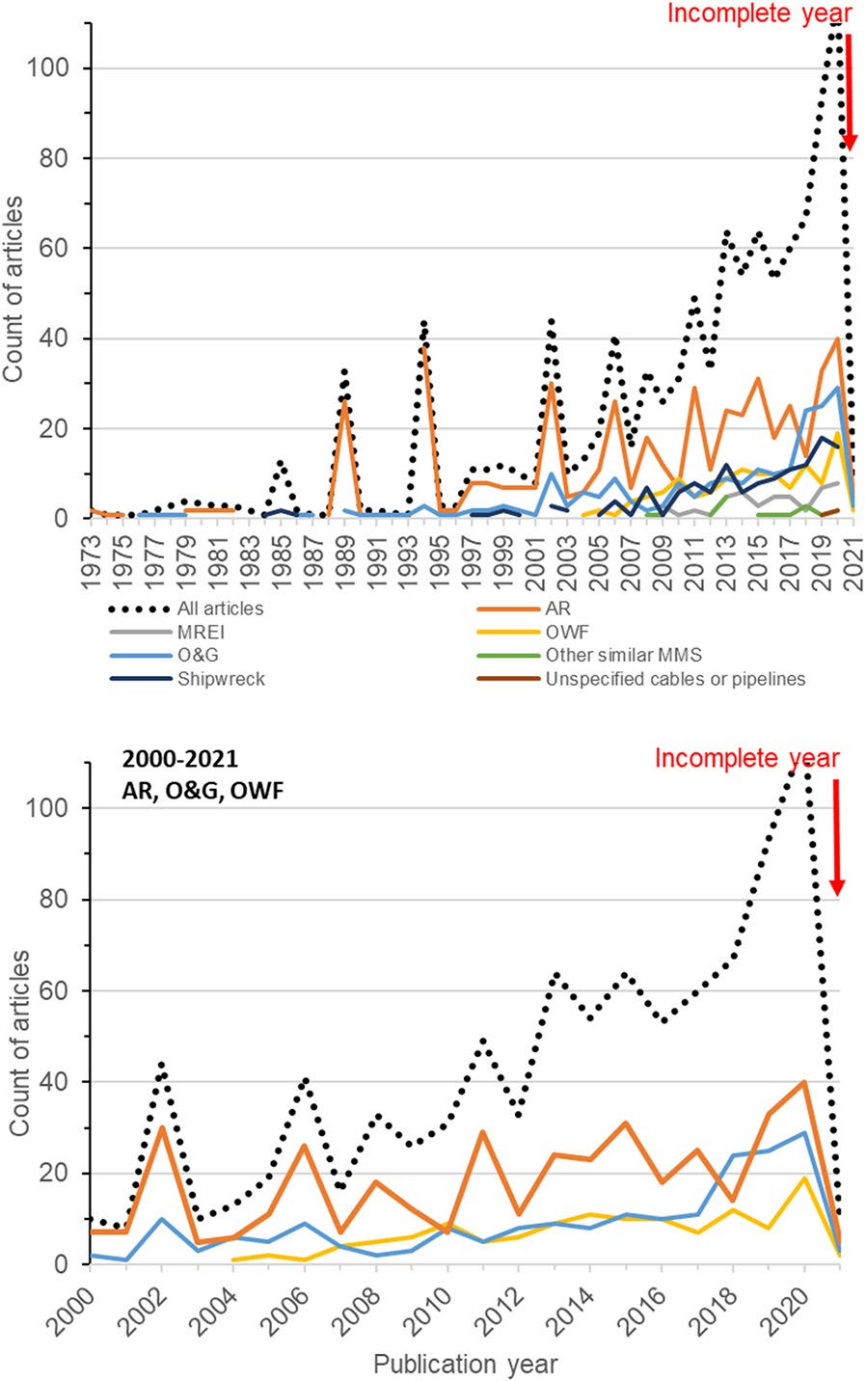
Count of articles over time for all MMS types (dotted line) and grouped by MMS type (coloured solid lines). Top: all MMS types and years. Bottom: close-up for the years 2000–2021 for selected MMS (oil and gas (O&G), offshore wind farms (OWF), and artificial reefs (AR)). Please note that the year 2021 was incomplete as searches ended in February 2021

There has been an increase in the number of articles published per annum related to the effects of MMS in the sea relevant to this map from the first article found (published in 1973) (Fig. 2 top). Although variable from year-to-year, the greatest number of articles published in a year was in 2020 (101)—the most recent full year to date. With the exception of 2010 and 2018, the majority of articles each year focuses on the effects of artificial reefs, although from 2018, there was a notable increase in the number of studies related to oil and gas (2018–24; 2020–29) and offshore wind farms (2018–12; 2020–19) (Fig. 2 bottom).

**Fig. 3:**
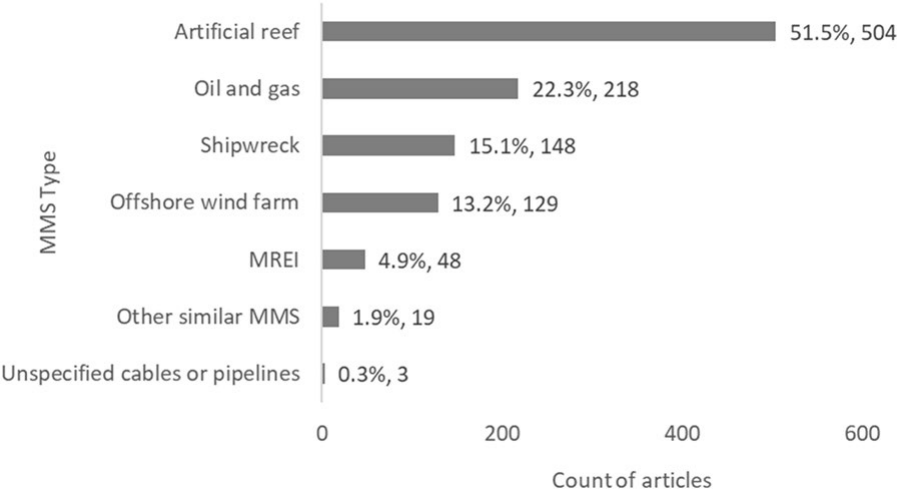
Count of articles by MMS type. MREI: Marine renewable energy installation. MMS: Man-made structure. Data labels at end of bars are percentages of total articles (N = 979), followed by exact counts. NB: The total count of articles may be greater than the number of unique articles, and percentages may add up to more than 100% as some articles contains studies spanning multiple MMS types

Overall, ARs are the focus of 51.5% (n = 504) of all articles included in the map, followed by O&G infrastructures (22.3%, n = 218), shipwrecks (15.1%, n = 148), and OWFs (13.2%, n = 129) (Fig. 3). MREIs, cables and pipelines, and other similar MMS represented a small percentage of all articles (4.9%, n = 48; 0.3%, n = 3; and 1.9%, n = 19, respectively). No articles were found relating to CCS.

### Distribution of evidence by country and geographical marine location

**Fig. 4:**
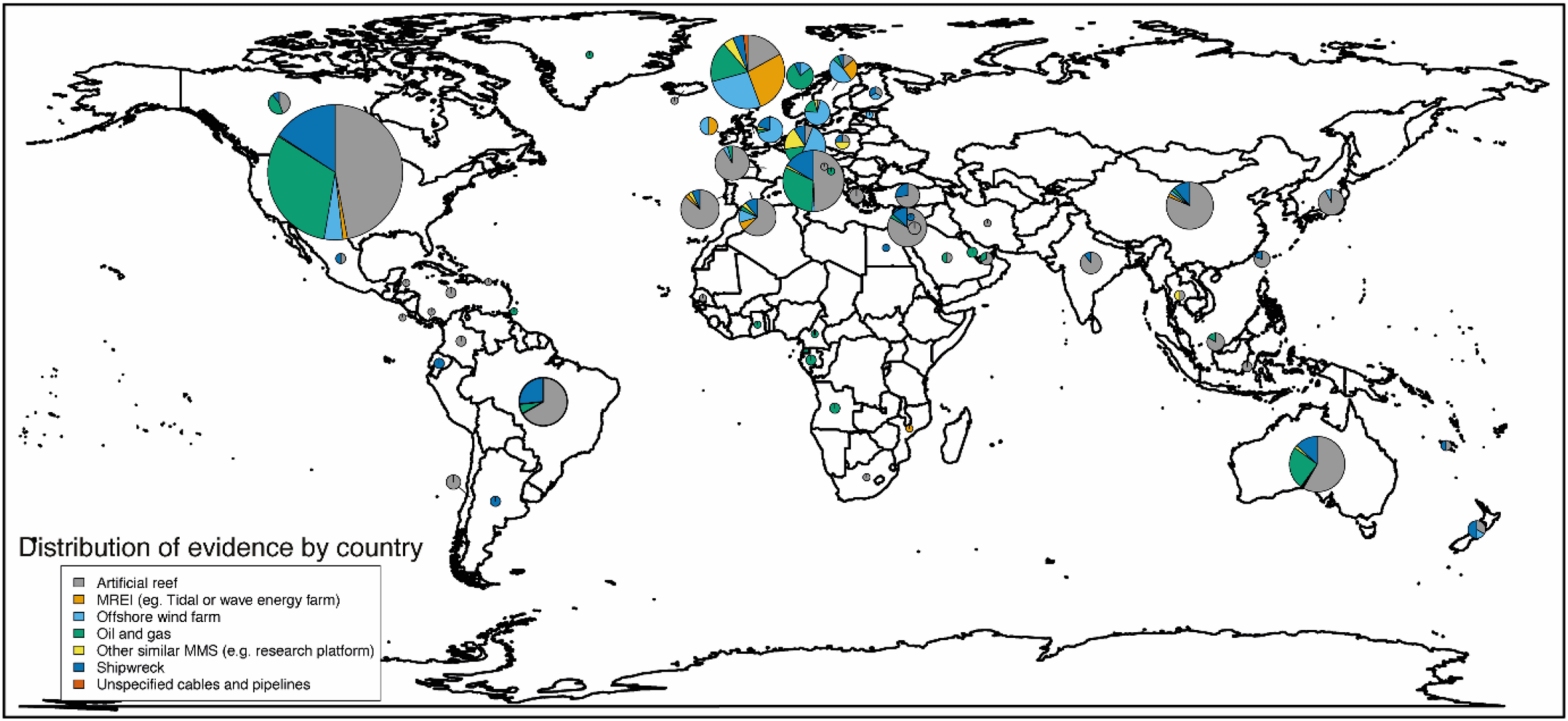
Geographical distribution of articles (by country), grouped by MMS type (excluding 25 ‘global or multiple countries’ studies not mapped here). The size of the pie chart is scaled relative to the number of articles associated with each country. Refer to Table 3 for details on the number of articles for each country

Articles describing the effects of MMS in the sea were found for studies undertaken in 78 countries worldwide (Table 3, Fig. 4, Additional file 7: Table S1), with the number of articles varying widely depending on the country the studies took place in. Articles disproportionately presented studies undertaken in the United States (313), the United Kingdom (97), Italy (68), and Australia (55). All other countries were the focus of studies published in fewer than 50 articles each. Additionally, 25 articles described studies with a ‘global’ geographical scope or spanning multiple (more than 6) countries. The geographical locations of studies presented in 3 articles were categorised as 'unclear or unspecified'.

**Table 3:** Distribution of articles by country

Country	Number of articles*
USA	313
UK	97
Italy	68
Australia	55
Brazil	43
China	39
Germany	29
Portugal	28
Israel	26
The Netherlands	26
*Global or multiple countries*^*‡*^	25
France	24
Denmark	23
Belgium	22
Spain	22
Norway	14
Sweden	14
Japan	12
Turkey	11
India	9
The Bahamas	9
Canada	8
South Korea	8
Malaysia	6
Ireland	5
Chile	4
Greece	4
New Zealand	4
Taiwan	4
Cyprus	3
Jordan	3
Maldives	3
The Philippines	3
*Unclear or unspecified*	3
Angola	2
Argentina	2
Cape Verde	2
Finland	2
Gabon	2
Indonesia	2
Jamaica	2
Mexico	2
Poland	2
Qatar	2
Saudi Arabia	2
Scotland	2
Singapore	2
Thailand	2
United Arab Emirates	2
Antigua	1
Barbados	1
Belize	1
Cameroon	1
Cayman Islands	1
Colombia	1
Costa Rica	1
Croatia	1
Curacao	1
Democratic Republic of Congo	1
Ecuador	1
Egypt	1
Equatorial Guinea	1
Estonia	1
Federated States of Micronesia	1
Ghana	1
Greenland	1
Guam	1
Iceland	1
Iran	1
Kiribati	1
Korea	1
Lebanon	1
Mozambique	1
New Caledonia	1
Palau	1
Panama	1
Puerto Rico	1
Senegal	1
Slovenia	1
South Africa	1
Trinidad and Tobago	1

*Grand total may be greater than the number of unique articles (N = 979), as some articles contains studies spanning multiple countries

^‡^Articles presenting studies with a global scope or spanning 6 or more countries

**Fig. 5:**
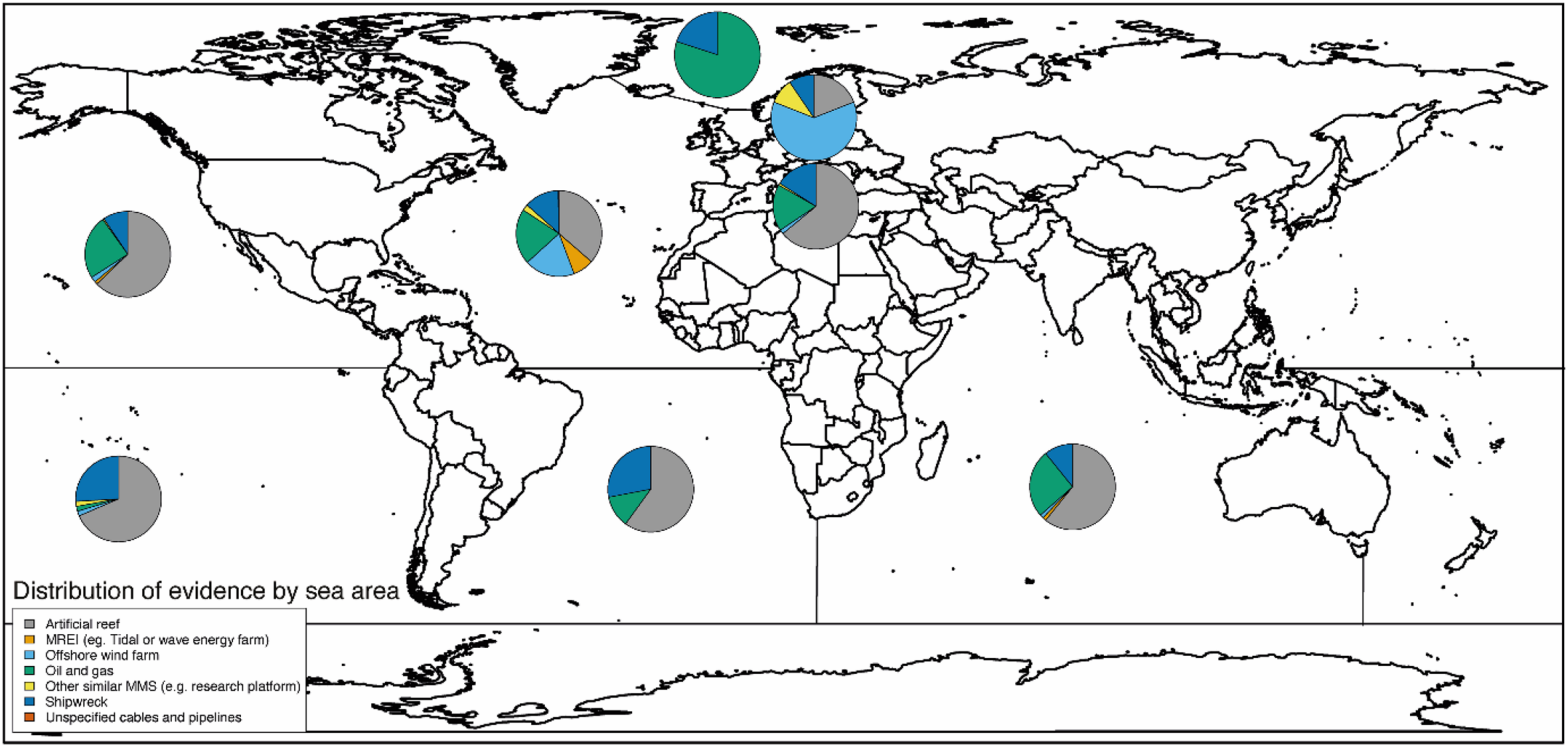
Geographical distribution of articles (by sea area), grouped by MMS type (excluding 12 ‘global’ studies and 2 ‘unclear or unspecified’ studies not mapped here). Sea areas represented: North Pacific Ocean, South Pacific Ocean, Arctic Ocean, North Atlantic Ocean (excl. Mediterranean Sea, excl. Baltic Sea), Baltic Sea, Mediterranean Sea, South Atlantic Ocean, Indian Ocean, Equatorial Pacific. Note that no studies were found for the Southern Ocean. The size of the pie chart is not scaled relative to the number of articles associated with each sea area. Refer to Table 4 for more details on the geographical spread of studies, by sea areas and sub-areas

The majority of articles (64.3%) were focused on the North Atlantic Ocean region (including the Gulf of Mexico (12.7%), the North Sea (12.5%) and the Mediterranean Sea (12.2%)) and the North Pacific Ocean region (17.6%) (Table 4, Fig. 5). The Indian Ocean accounted for 6.7% of articles. The South Atlantic and the South Pacific Oceans were the focus of 4.7% and 4.3% of articles, respectively. Just 0.5%, 0.8% and 0.5% of articles focused on the equatorial Atlantic, equatorial Pacific, and Arctic Oceans, respectively, and no articles were returned for the Southern Ocean. 12 articles had a global oceanic scope, and 2 had an unclear or unspecified scope.

**Table 4:** Distribution of articles by regional oceanic regions

Geographical location – regional ocean	Count of unique articles	Percentage of total unique articles*
*North Atlantic Ocean*	*629*	*64.25%*
North-east Atlantic Ocean	142	14.50%
North Sea	122	12.46%
Mediterranean Sea	119	12.16%
North-west Atlantic Ocean	122	12.46%
Gulf of Mexico	124	12.67%
*South Atlantic Ocean*	*46*	*4.70%*
South-west Atlantic Ocean	43	4.39%
South-east Atlantic Ocean	3	0.31%
*Equatorial Atlantic*	*4*	*0.40%*
*North Pacific Ocean*	*172*	*17.57%*
North-east Pacific Ocean	91	9.30%
North-west Pacific Ocean	81	8.27%
*South Pacific Ocean*	*43*	*4.39%*
South-east Pacific Ocean	5	0.51%
South-west Pacific Ocean	38	3.88%
*Equatorial Pacific*	*8*	*0.82%*
*Indian Ocean*	*66*	*6.74%*
*Arctic Ocean*	*5*	*0.51%*
*Southern Ocean*	*0*	*0.00%*
Global	12	1.23%
Unclear or Unspecified	2	0.20%
Grand Total*	988	

*Grand total may be greater than the number of unique articles (N = 979), and percentages may add up to more than 100% as some articles contains studies spanning multiple oceanic areas

### Distribution of evidence by population classification

**Fig. 6:**
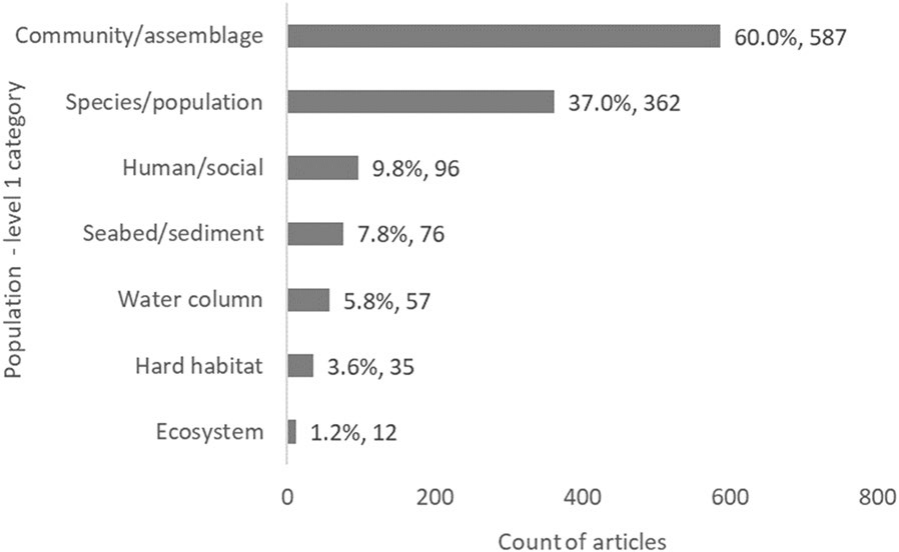
Count of articles by Population—level 1 category. Data labels at end of bars are percentages of total articles (N = 979), followed by exact counts. NB: The total count of articles may be greater than the number of unique articles, and percentages may add up to more than 100% as some articles contains studies spanning multiple Population categories

The number of articles relating to a particular Population—level 1 (e.g. community/assemblage, species/population, human/social, etc.) was highly variable. Articles presented studies relating in the vast majority to the following Population (level 1) categories: community/assemblages (60%, 587 articles), followed by species/populations (37%, 362), and human/social (9.8%, 96). All other Population (level 1) categories were represented in fewer than 10% of all articles (Fig. 6).

When looking closer at the spread of evidence for each Population (level 1) category by MMS type (Table 5), clear clusters and gaps are apparent. There seems to be a bias towards investigating the effects of artificial reefs on communities or assemblages (330 articles), as well as species or populations (177). Knowledge clusters also exist for the effects of oil and gas infrastructures on communities/assemblages and species/populations (139 and 87, respectively), the effects of shipwrecks on communities/assemblages (94), and the effects of offshore wind farms on species/populations (62). All other Population (level 1) components were the focus of fewer than 60 studies. Knowledge gaps are particularly evident for whole ecosystems and hard habitats, regardless of the MMS type (although to a lesser extent for artificial reefs).

**Table 5:**
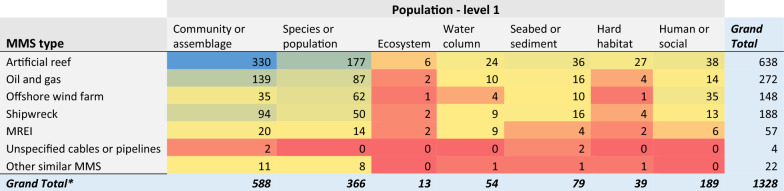
Heatmap illustrating the distribution of evidence from studies reporting on Population data (level 1), grouped by MMS type

Darker bluecells indicate most studies; while red cells indicate fewest studies

*Numbers can exceed the total number of articles (N = 979) in the map due to multiple Population and MMS components being reported within a single study

**Fig. 7:**
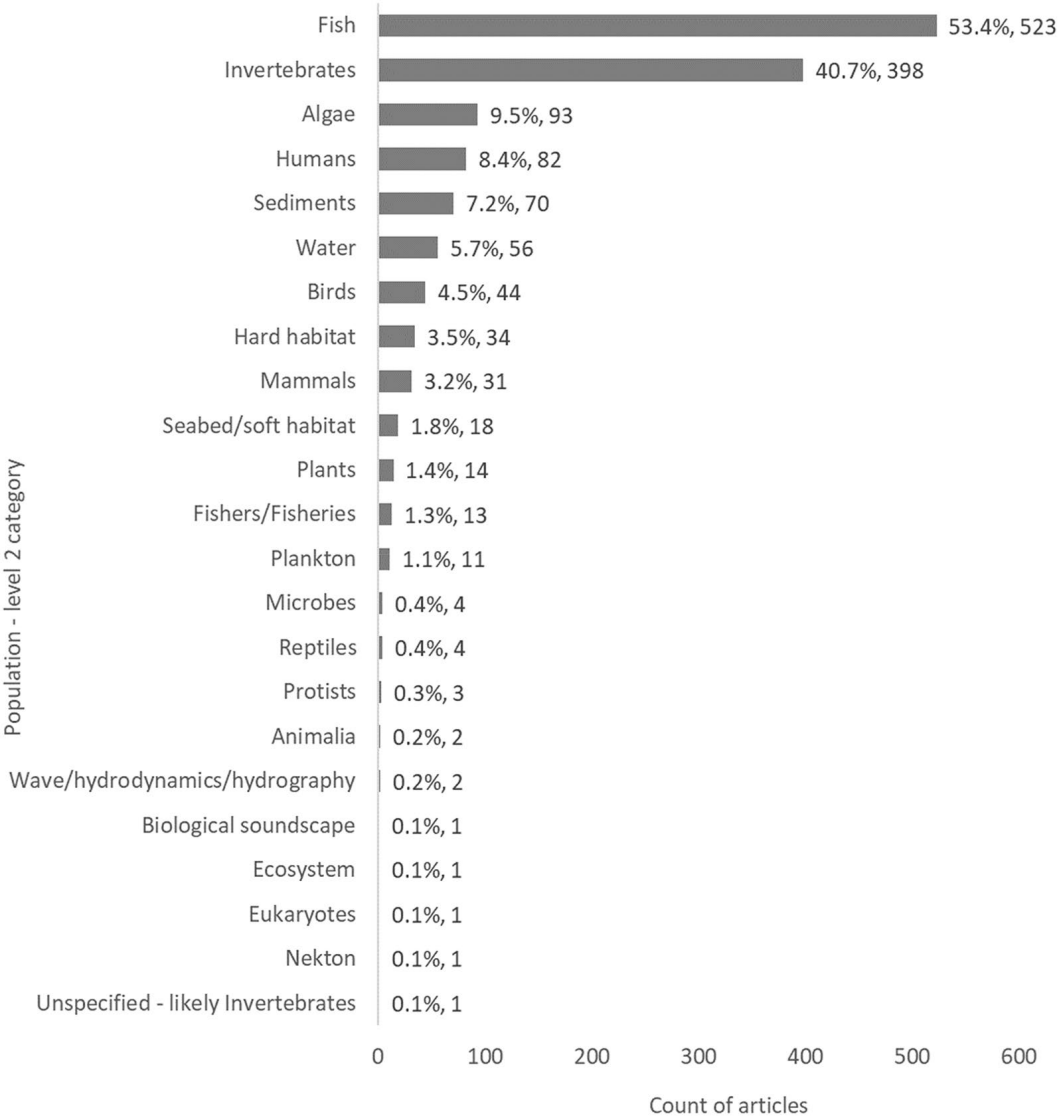
Count of articles by Population—level 2 categories. Data labels at end of bars are percentages of total articles (N = 979), followed by exact counts. NB: The total count of articles may be greater than the number of unique articles (N = 979), and percentages may add up to more than 100%, as some articles contains studies spanning multiple Population—level 2 categories (e.g.: studies on both Fish and Invertebrates)

When focussing on the spread of evidence relating to sub-categories of Populations (Population—level 2), a disproportionate number of studies were focused on either fish (54%, 523 articles) or invertebrates (42%, 398), whilst the remaining classification categories were typically represented by fewer than 10% of articles (Fig. 7). Similarly, there appears to be clear knowledge clusters relating to artificial reefs when it comes to these Population (level 2) components (fish: 327 articles, invertebrates: 199), as well as oil and gas to a lesser extent (fish: 125, invertebrates: 94) (Table 6).

**Table 6:**
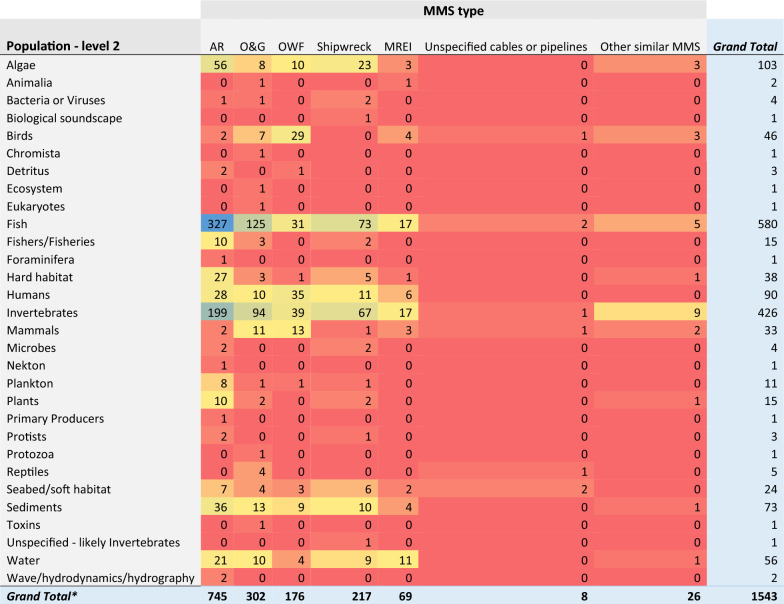
Heatmap illustrating the distribution of evidence from studies reporting on Population data (level 2), grouped by MMS type

Darker bluecells indicate most studies; while red cells indicate fewest studies. *Numbers can exceed the total number of articles (N = 979) in the map due to multiple Population and MMS components being reported within a single study. One article for which the Population – level 2 was unspecified was omitted from this heatmap

### Distribution of evidence by outcome classification

**Fig. 8:**
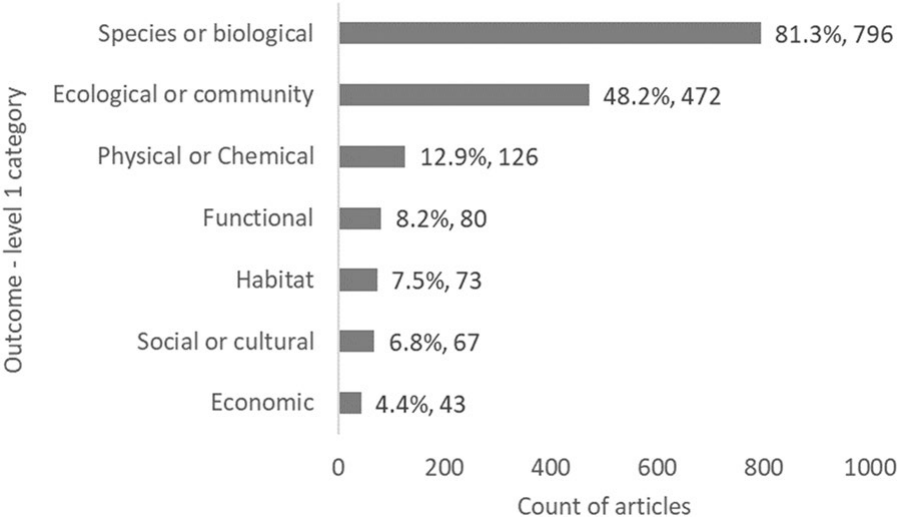
Count of articles by Outcome—level 1 category. Data labels at end of bars are percentages of total articles (N = 979), followed by exact counts. NB: The total count of articles may be greater than the number of unique articles, and percentages may add up to more than 100% as some articles contains studies spanning multiple Outcome categories

Species/biological and ecological/community Outcomes (level 1) are the focus of most articles (81.3%, 796 articles, and 48.2%, 472 articles, respectively), with otherwise minimal attention on physical/chemical outcomes (12.9%, 126 articles) and other outcomes (social cultural, habitat, functional, and economic, all < 10% of articles) (Fig. 8).

When looking closer at the spread of evidence for each Outcome (level 1) category by MMS type (Table 7), again clear clusters and gaps are apparent. There seems to be a bias towards investigating the effects of artificial reefs on biological (432 articles) and ecological (277) outcomes, while other Outcome components (functional, habitat, and economic) were the focus of much fewer studies, particularly when considering functional, habitat, and economic outcomes for OWFs, MREIs or shipwrecks, which each counted fewer than 16 articles.

**Table 7:**
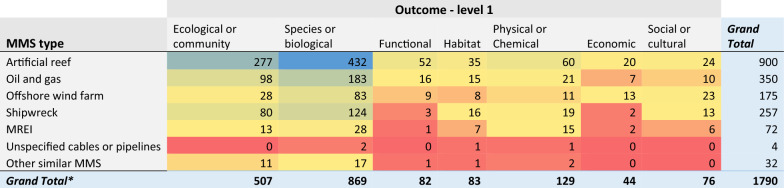
Heatmap illustrating the distribution of evidence from studies reporting on Outcome data (level 1), grouped by MMS type

Darker bluecells indicate most studies; while red cells indicate fewest studies. *Numbers can exceed the total number of articles (N = 979) in the map due to multiple Outcome and MMS components being reported within a single study

When focussing on the spread of evidence relating to sub-categories of Outcomes (Outcome—level 2), a disproportionate number of studies investigated species or population’s abundance, density, or percentage cover (66.1% of articles, 647 articles), diversity metrics (38.4%, 376), community composition or community structure (33.2%, 325), organisms’ size, growth, or age (20.5%, 201), organisms or population biomass (20.2%, 198), the remaining outcome metrics were, with the exception of species or population’s range, distribution, or larval dispersal (14.5%, 142), typically represented by fewer than 100 articles (~ 10)% of all articles (Table 8).

**Table 8:** Counts and percentages of unique articles by Outcome—level 2 category

Outcome *level 1*—level 2	Count of unique articles		Percentage of unique articles*
*Species or biological*	*796*	*81.31%*	
Abundance, density, or % cover	647	66.09%	
Size/growth, or age	201	20.53%	
Biomass	198	20.22%	
Range or distribution or larval dispersal/connectivity	142	14.50%	
Behaviour (reproductive, avoidance, migration, use)	92	9.40%	
Fecundity, Reproductive output (incl. fertility, hatchling success), egg/sperm quality, or recruitment	46	4.70%	
Survival/mortality	35	3.58%	
Condition, health, or injury	27	2.76%	
Sex ratio	19	1.94%	
Genetic (allele frequency, connectivity, genotype/phenotpy, genetic variation)	9	0.92%	
*Ecological or community*	*472*	*48.21%*	
Diversity (H, J, Δ, richness, genetic)	379	38.41%	
Community composition or structure	325	33.20%	
Trophic structure	48	4.90%	
Species interaction	7	0.72%	
*Physical or Chemical*	*126*	*12.87%*	
Biochemistry (Chl-a, nutrients, metals…)	62	6.33%	
Sediment type or grain size	47	4.80%	
Hydrodynamics	27	2.76%	
Sedimentation	26	2.66%	
Temperature, light, salinity, dissolved oxygen…	19	1.94%	
Abundance or biomass of plastics or particles	1	0.10%	
*Functional*	*80*	*8.17%*	
Predation, Herbivory, or Diet composition	58	5.92%	
Productivity	23	2.35%	
Biological Trait Analysis	1	0.10%	
*Habitat*	*73*	*7.46*	
Habitat quantity, quality or extant (artificial)	45	4.60%	
Habitat quantity, quality or extant (natural)	35	3.580%	
*Social or cultural*	*67*	*6.84%*	
Other attitude, perception, or value metrics	49	5.01%	
Frequency, duration or rates of visits (e.g. recreation or tourism)	16	1.63%	
Injury, fatality, or other human harm	2	0.20%	
Preferences (e.g. using photos, geo-tagged photos, or social media data)	1	0.10%	
*Economic*	*43*	*4.39%*	
Financial gain or loss (individuals or organisations)	26	2.66%	
Willingness to pay, travel time–cost estimate, or other related metrics	16	1.63%	
Incident/accident	2	0.20%	
Material or equipment loss (e.g. vessel, gear…)	1	0.10%	
Grand Total (level 2)*	1,657		

*Grand total may be greater than the number of unique articles (N = 979), and percentages may add up to more than 100%, as some articles contains studies spanning multiple Outcome categories

### Distribution of evidence by study design

The majority of articles were empirical or observational studies (832; 85%) undertaken either as control-impact, reference site or site comparison studies (spatial comparator; 50.8% of all articles) (Table 9). Modelling studies accounted for 9% (88), qualitative or quantitative social studies for 7% (68), and meta-analyses (with or without systematic reviews) cumulatively making up the remaining 0.8% (8) (Table 2). A striking number of studies did not include any type of comparator (27.9% of all articles). Studies using 'Before-After' (BA; temporal comparator) or 'Before-After-Control-Impact' (BACI; temporal and spatial comparators) designs that are required under Environmental Impact Assessment (EIA) legislation were relatively infrequent (4.4% and 3.3% of all studies, respectively).

**Table 9:** Counts and percentages of articles by Study design

Study design *level 1*- level 2	Count of unique articles	Percentage of unique articles within level 1	Percentage of total unique articles*
*Empirical or observational study*	*832*		*84.98%*
Control-impact, reference site or site comparison	423	50.84%	43.21%
No comparator	247	29.69%	25.23%
After only study (multiple time points/succession)	146	17.55%	14.91%
Before-after study	38	4.57%	3.88%
Before-after-control-impact study	29	3.49%	2.96%
Correlative only (no direct effects)	15	1.80%	1.53%
*Modelling study*	*88*		*8.99%*
Scenarios comparisons	32	36.36%	3.27%
No comparator	26	29.55%	2.66%
Correlative only (no direct effects)	13	14.77%	1.33%
Control-impact, reference site or site comparison	8	9.09%	0.82%
Before-after study	5	5.68%	0.51%
After only study (multiple time points/succession)	4	4.55%	0.41%
Bayesian networks (Belief networks)	2	2.27%	0.20%
Before-after-control-impact study	2	2.27%	0.20%
*Qualitative or quantitative social study*	*68*		*6.95%*
Survey/questionnaire	44	64.71%	4.49%
Interviews	18	26.47%	1.84%
Participatory mapping	2	2.94%	0.20%
Social media/web scraping	1	1.47%	0.10%
Frequency, duration, or rates of visits	1	1.47%	0.10%
Focus group/workshop/deliberative	1	1.47%	0.10%
Other attitude, perception, or value metrics	1	1.47%	0.10%
*Systematic review and meta-analysis*	*5*		*0.51%*
Control-impact, reference site or site comparison	4	80.00%	0.41%
Before-after-control-impact study	1	20.00%	0.10%
*Meta-analysis only*	*3*		*0.31%*
After only study (multiple time points/succession)	1	33.33%	0.10%
Scenarios comparisons	1	33.33%	0.10%
Control-impact, reference site or site comparison	1	33.33%	0.10%
Grand Total (level 2)*	1,066		

*Grand total may be greater than the number of unique articles (N = 979), and percentages may add up to more than 100%, as some articles contains studies spanning multiple study designs

Within empirical or observational studies, the majority incorporated a comparator undertaken either as control-impact, reference site or site comparison studies (as mentioned above), or as an ‘After-only’ study (temporal comparator—successional studies; 17.6%). Fewer involved a BA design (4.6%) or a BACI design (3.5%). Modelling studies mostly involved scenario comparisons (36.4% of modelling studies), while social studies designs were largely based on surveys and questionnaires (64.7% of social studies).

### Distribution of evidence related to decommissioning

**Fig. 9:**
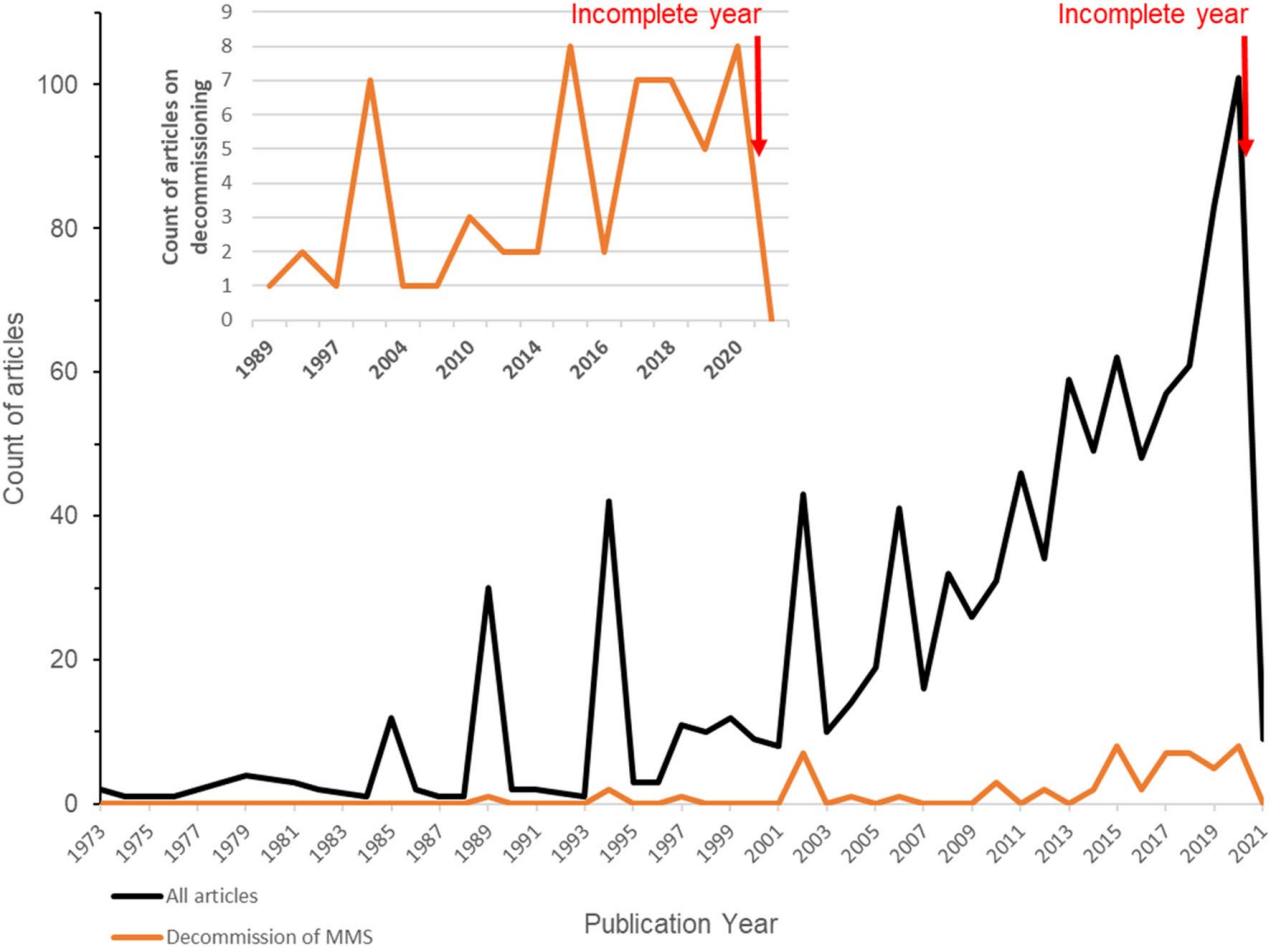
Number of articles published per year on the effects of marine MMS, showing both all-Exposure types combined (black line) and articles specifically on decommissioning (orange line). Insert: Close-up for the years 1989–2021 on the number of articles on decommissioning of MMS. Please note the difference in vertical scale between the two graphs. Please note that the year 2021 was incomplete as searches ended in February

Articles reporting on evidence for the effects of decommissioning of MMS – the focus of our second priority objective – were nearly non-existent prior to 2002 when a first publication peak is noticeable (Fig. 9). This is in part due to a special issue of *ICES Journal of Marine Science* on “Rigs-to-Reefs” published at the time. The number of articles on decommissioning started increasing in the years 2010–2020 to a maximum of 8 annually in both 2015 and 2020, which nevertheless remains noticeably low (Fig. 9).

**Fig. 10:**
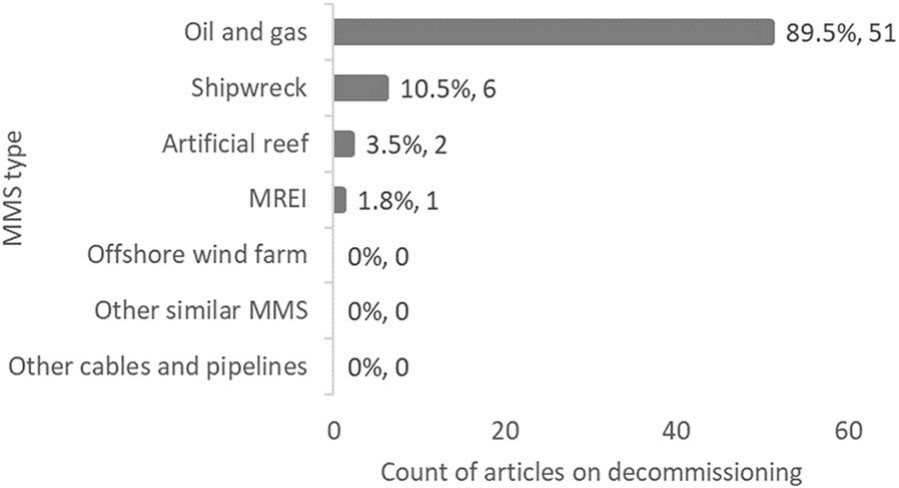
Count of articles on decommissioning by MMS type. NB: The total count of articles may be greater than the number of unique articles (N = 57), and percentages may add up to more than 100%, as some studies investigated more than one category of MMS

Unsurprisingly, decommissioning studies are mostly related to O&G installations (51 out of the 57 articles), but some relate to shipwrecks (6), ARs (2), and MREI (1) (Fig. 10). None have been identified about OWFs, cables and pipelines, nor about other MMS types.

**Fig. 11:**
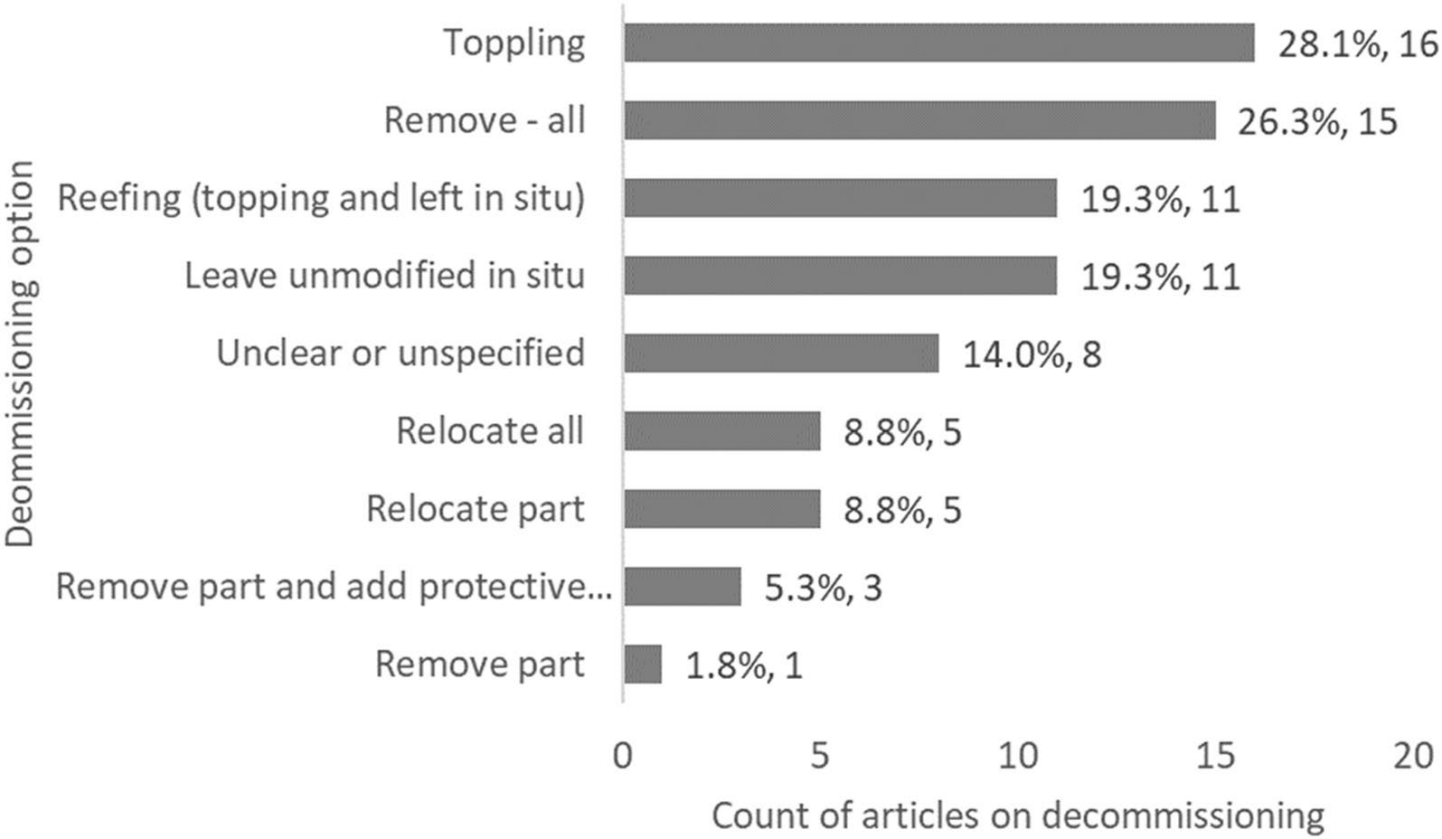
Count of articles on decommissioning grouped by decommissioning exposure option. Data labels at end of bars are percentages of total articles (N = 57), followed by exact counts. NB: The total count of articles may be greater than the number of unique articles, and percentages may add up to more than 100%, as some studies investigated more than one option

When looking at the decommissioning method, toppling and complete removal were the most studied (16 and 15 articles each, respectively), followed by reefing (topping) and leaving unmodified in situ (11 articles each) (Fig. 11). Other methods were less studied. Studies presented in 8 articles had either unclear or unspecified decommissioning methodologies.

**Fig. 12:**
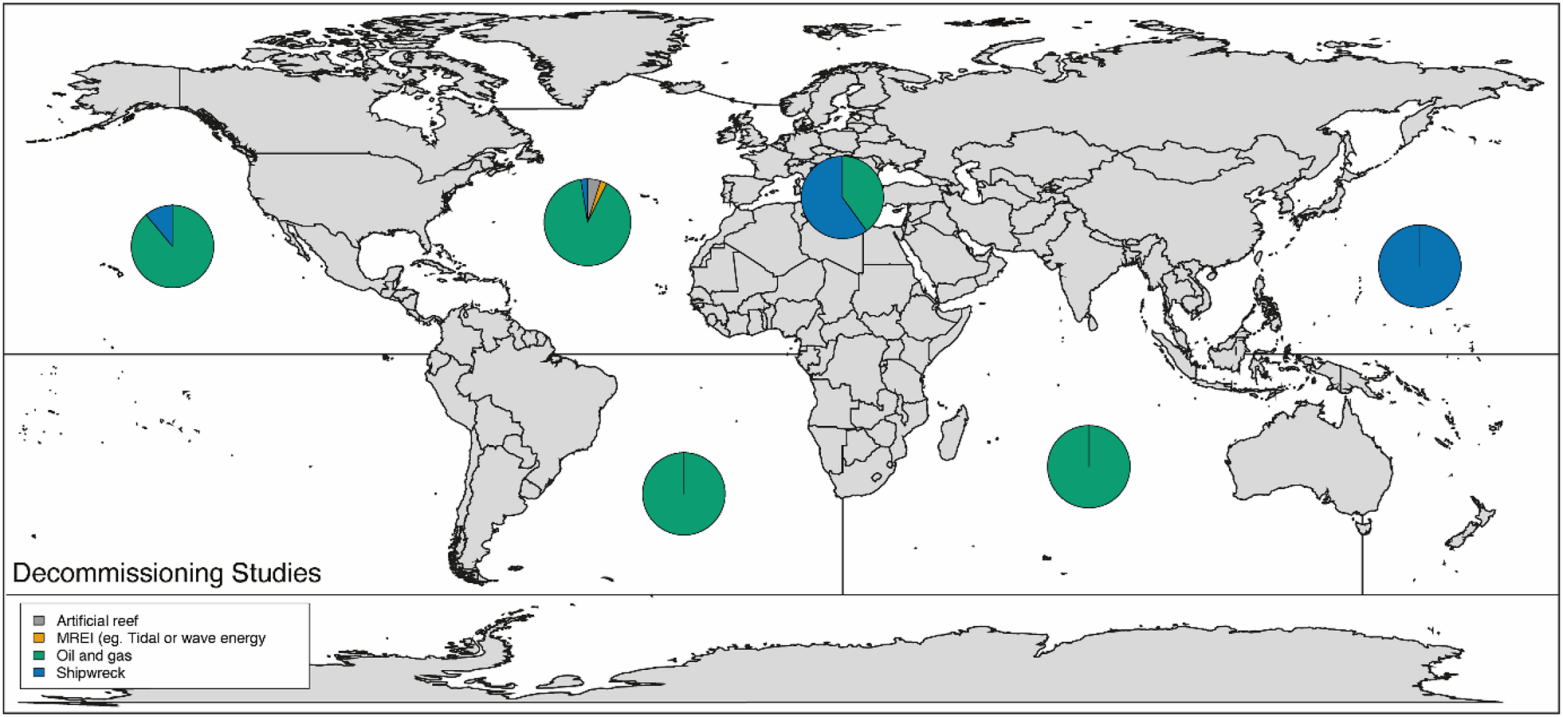
Geographical distribution of articles on decommissioning (by sea area), grouped by MMS type (N = 57)*.* Sea areas represented: North Pacific Ocean, North Atlantic Ocean (excl. Mediterranean Sea, excl. Baltic Sea), Mediterranean Sea,, South Atlantic Ocean, Indian Ocean, Equatorial Pacific. Note that no studies were found for the Arctic Ocean, the Baltic Sea, the Equatorial Atlantic, the South Pacific Ocean, and the Southern Ocean. The size of the pie chart *is not* scaled relative to the number of articles associated with each sea area. Refer to Table 11 for more details on the geographical spread of studies, by sea areas and sub-areas

**Fig. 13:**
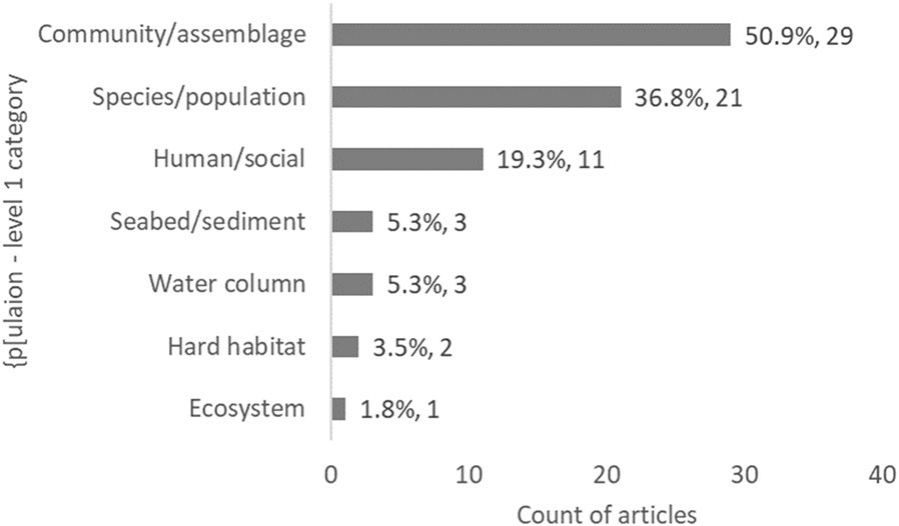
Count of articles on decommissioning by Population—level 1 category. Data labels at end of bars are percentages of total articles (N = 57), followed by exact counts. NB: The total count of articles may be greater than the number of unique articles, and percentages may add up to more than 100%, as some articles contains studies spanning multiple Population categories

**Fig. 14:**
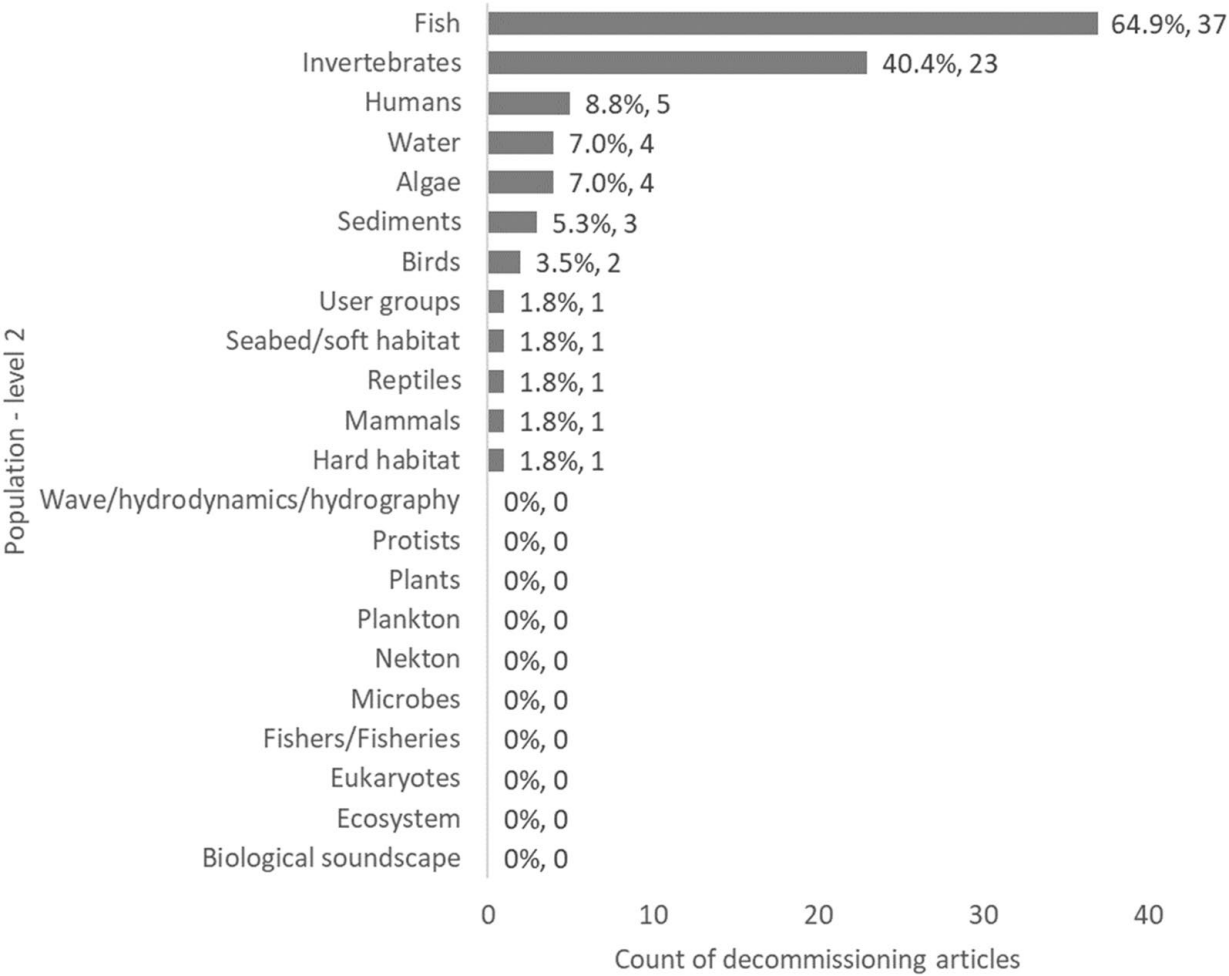
Count of articles on decommissioning by Population—level 2 category. Data labels at end of bars are percentages of total articles (N = 57), followed by exact counts. NB: The total count of articles may be greater than the number of unique articles, and percentages may add up to more than 100%, as some articles contains studies spanning multiple Population—level 2 categories (e.g.: studies on both Fish and Invertebrates). Categories with a count of zero are based on categories found in other (non-decommissioning) articles included in the map

Studies were mostly undertaken in the USA (35 articles—again, unsurprisingly due to their “Rigs-to-Reef programmes), and north-west Europe (UK: 6, Italy: 5, Norway: 4, The Netherlands: 3), with all other countries being the focus of 2 or fewer articles (Table 10). Consequently, most studies were located in the Gulf of Mexico (43.9%), the north-east Pacific Ocean (15.8%), and the North Sea (17.5%) (Table 11).

**Table 10:** Geographic spread of MMS decommissioning studies

Country of study	Count of articles on decommissioning
USA	35
UK	6
Italy	5
Norway	4
The Netherlands	3
*Global or multiple countries*^*‡*^	2
Australia	2
Brazil	1
Sweden	1
Canada	1
Croatia	1
Malaysia	1
Germany	1
Denmark	1
Grand Total*	64

*Grand total may be greater than the number of unique articles (N = 57), as some articles contains studies spanning multiple countries

^‡^Articles presenting studies with a global scope or spanning 6 or more countries

**Table 11:** Distribution of articles on MMS decommissioning by regional oceanic regions

Geographical location (level 2)	Count of articles on decommissioning	Percentage of total unique articles
*North Atlantic Ocean*	43	75.43%
Gulf of Mexico	25	43.86%
North Sea	10	17.54%
Mediterranean Sea	5	8.77%
North-east Atlantic Ocean	2	3.51%
North-west Atlantic Ocean	1	1.75%
*South Atlantic Ocean*	2	3.50%
South-west Atlantic Ocean	1	1.75%
South-east Atlantic Ocean	1	1.75%
*Equatorial Atlantic Ocean*	0	0%
*North Pacific Ocean*	9	15.79%
North-east Pacific Ocean	8	14.04%
North-west Pacific Ocean	1	1.75%
*South Pacific Ocean*	0	0%
South-east Pacific Ocean	0	0%
South-west Pacific Ocean	0	0%
*Equatorial Pacific Ocean*	1	1.75%
*Indian Ocean*	2	3.51%
*Arctic Ocean*	0	0%
*Southern Ocean*	*0*	*0%*
Global	0	0%
Grand Total	57	

**Fig. 15:**
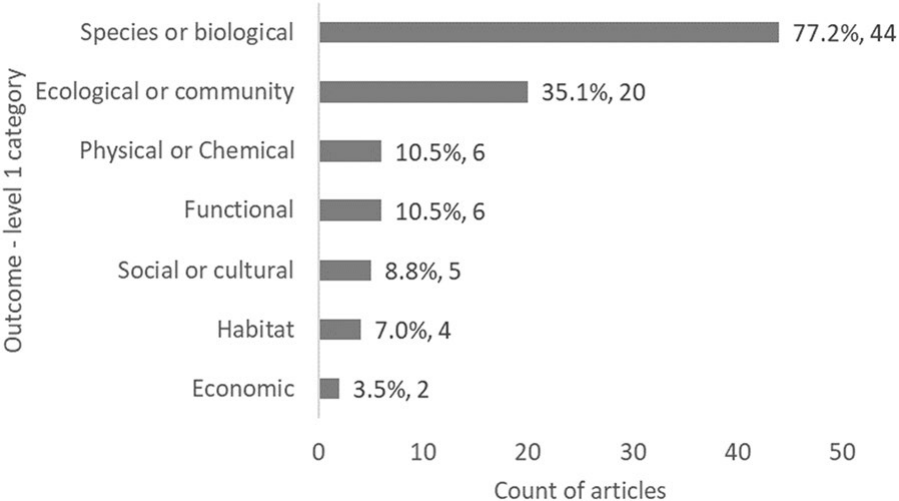
Count of articles on decommissioning by Outcome—level 1 category. Data labels at end of bars are percentages of total articles (N = 57), followed by exact counts. Percentages may add up to more than 100% as some articles contains studies spanning multiple Outcome categories

The studies focussed particularly on the following Population (level 1): communities and assemblages (29 articles; 50.9%) and species and populations (21 articles; 36.8%), but also on human and social aspects (11 articles; 19.3%) (Fig. 12), with a focus on fish (37 articles) and invertebrates (23 articles) as Population level 2 (Fig. 13). Given the Population focus, study Outcomes were hence related in majority to species or biological metrics (44 articles; 77.2%; such as abundance 49.1%) and ecological or community metrics (20 articles; 35.1%; such as diversity 33.3%) (Figs. 14 and 15, Table 12).

**Table 12:** Counts and percentages of articles on decommissioning by Outcome—level 2 category

Outcome categories (*level 1*–level 2)		Count of articles on decommissioning		Percentage of articles on decommissioning*
*Species or biological*		*44*		*77.19%*
Abundance, density, or % cover		28		49.12%
Biomass		18		31.58%
Condition, health, or injury		2		3.51%
Fecundity, Reproductive output (incl fertility, hatchling success), egg/sperm quality, or recruitment		3		5.26%
Genetic (allele frequency, connectivity, genotype/phenotpy, genetic variation)		2		3.51%
Range or distribution or larval dispersal/connectivity		7		12.28%
Sex ratio		1		1.75%
Size/growth, or age		17		29.82%
Survival/mortality		2		3.51%
*Ecological or community*		*20*		*35.09%*
Community composition or structure		14		24.56%
Diversity (H, J, Δ, richness, genetic)		19		33.33%
Trophic structure		3		5.26%
*Functional*		*6*		*10.53%*
Predation, Herbivory, or Diet composition		4		7.02%
Productivity		2		3.51%
*Physical or Chemical*		*6*		*10.53%*
Biochemistry (chl-a, nutrients, metals…)		2		3.51%
Hydrodynamism		1		1.75%
Sedimentation		3		5.26%
*Habitat*		*4*		*7.02%*
Habitat quantity, quality or extant (artificial)		1		1.75%
Habitat quantity, quality or extant (natural)		3		5.26%
*Social or cultural*		*5*		*8.77%*
Frequency, duration or rates of visits (e.g. recreation or tourism)		1		1.75%
Other attitude, perception or value metrics		4		7.02%
*Economic*		*2*		*3.51%*
Financial gain or loss (individuals or organisations)		2		3.51%
Grand Total (level 2)*		139		

*Grand total may be greater than the number of unique articles (N = 57), and percentages may add up to more than 100%, as some articles contains studies spanning multiple Outcome categories

### Limitations of the map

The search strategy employed to generate this map was designed to capture the breadth of relevant topics, but it is not exhaustive. We have captured a large number of articles (979) relevant to the questions surrounding decommissioning of MMS, but also recognise that there are numerous articles (n = 1252) that have been excluded. The majority were excluded on the basis that they present concepts, frameworks or reviews of other evidence rather than providing new empirical evidence/data per se. Despite the number of returned articles, there was a risk that our search terms were still too narrow, and studies using uncommon synonyms could have been missed. This risk, which was inevitable given the breadth of our map, encompassing ecological, economic and social science disciplines, was initially pre-emptively mitigated by having both our internal Review Team and external, independent experts from our Stakeholder Group review the search terms. Nevertheless, it was clear from our ‘snowballing’ exercise that some risk of overlooking relevant peer-reviewed literature had remained; although this exercise (i.e. snowballing) helped mitigate it to some extent by retrieving a further 119 articles that had been initially missed with our search terms. Further search-string evaluation prompted by a reviewer during the peer-review process revealed that the term “renewable*” had been omitted and would have identified a non-negligible number of additional articles. This omission would likely have led to additional articles specifically on marine renewables being missed.

We also recognise that much of the environmental evidence of 'impact' of MMS may be contained in other types of publications, most notably the grey literature that may be commissioned by industry or government agencies and subsequently used in decision-making. This other source of literature may include information important to decision-making and policy formulation. However, our decision to focus on just the primary literature was intentional as our experience from the UK is that this evidence base is not being used to its full potential and currently makes a limited (if any) contribution to evidence-based decision-making. As such, the motivation of this systematic map was firstly to provide a review of worldwide published and peer-reviewed scientific evidence relevant to decommissioning, and secondly to identify gaps in knowledge. Its aim is therefore to provide the basis of future reinforcement or juxtaposition to the current evidence base and best practices to support the best ecological, social and economic outcomes following decommissioning of MMS.

Finally, as evidenced in this work (see also Fig. 2), research into the effects of marine MMS as well as decommissioning is an active and fast-paced one, which has been gaining momentum in recent years (see recent work by [54]). Thus, since undertaking our initial searches in spring 2021, additional relevant studies are likely to have been published, and thus missed in this work (such as [54] mentioned above). At the time of writing, a quick (and likely unreproducible) Google Scholar search for articles from late 2021/early 2022 with the key words “marine”, “structure”, and “decommissioning” retrieved several relevant hits, such as [55, 56], and [57], and using our search string retrieved further potentially relevant articles (including [58, 59], and [60]). Given the time and resources systematic mapping work takes, this was unavoidable. Readers should thus keep in mind when consulting our map that additional recent articles may exist but not be included (yet). Future updates to this systematic map would benefit from updated searches that will identify recent publications, but also include potential additional terms to the search string, such as “renewable*”.

## Conclusions

This systematic map, the very first of its kind, provides an overview of the existing evidence on the ecological and socio-economic effects of the presence of operational, altered or decommissioned man-made structures (MMS) in the sea from across the globe. We identified 979 articles across 3 types of MMS exposure, 7 main population categories, and 7 main outcome measures. Temporal trends revealed an exponential increase in the number of published studies per year from 1973 to date, the majority of which focused on AR structures, although since 2000, there has also been a burgeoning of literature on O&G infrastructure, OWFs and shipwrecks. The vast majority of literature considers the presence of MMS, but less than 6% of studies address issues related to their decommissioning, revealing a significant gap in evidence needed to underpin decision-making related to decommissioning options of MMS. The implications of this gap are explored below.

### Implication for policy/management

The processes of decommissioning MMS in the UK and internationally are regulated under global and regional instruments (see [61] for review). Yet, the evidence used in key decision-making processes, including licencing consent (e.g., Environmental Impact Assessment) and decommissioning, may lack sufficient peer-review or quantitative assessment [41] and/or may represent carefully chosen scenarios to suggest a low degree of environmental risk, thereby facilitating passing of licencing requirements and development (see [42] for discussion). Indeed, policy and decision-makers often use evidence presented in non peer-reviewed grey literature (such as industry-contracted research reports). While those sources may be valuable in their own right, they unlikely adhere to the principles described in [62], especially with respect to rigor, transparency and accessibility (e.g., unpublished, not peer-reviewed, limited circulation), which may in turn reduce the transparency of what evidence has been used in decision-making and how.

There is a pressing need for an objective, robust and quantitative assessment of the evidence to determine the general effects of MMS in the sea, and options for decommissioning, both globally and regionally. This is especially important in certain regions, for instance in the North Sea given recent changes to OSPAR Decision 98/3 on the Disposal of Disused Offshore Installations (1998). This, in combination with OSPAR Agreement 2012/13 (Guidelines on Artificial Reefs in relation to Living Marine Resources), all but prohibited the 'dumping, and leaving wholly or partly in place, of disused offshore installations'. This decision has subsequently been amended to state that a "competent authority of the relevant Contracting Party may give permission to leave installations or parts of installations in place in the case of: steel installations (> 10,000 tonnes in air); gravity-based concrete installations; floating concrete installations; or any concrete anchor-bases which results, or is likely to result in interference with other legitimate uses of the sea" (https://www.ospar.org/work-areas/oic/installations). There is widespread acceptance that complete removal may not be most beneficial [17], and that the repurposing of structures for socio-economic or environmental benefit (e.g., "Rigs-to-Reefs" programme in the USA, [16]) can be achieved, despite the limited direct evidence available, which has been highlighted in this systematic map (only 16 relating to ‘decommissioning—toppling’, 15 articles relating to ‘decommissioning—remove all’, and 11 relating to ‘decommissioning—reefing’). Nevertheless, the benefits of either full removal or repurposing should be weighed against the possible negative consequences of leaving structures unmodified in place, including their potential to act as stepping-stones for dispersal and facilitating the spread of non-native invasive species e.g. [63–66] (66 articles in this map mention non-native species to an extent). Although providing limited direct evidence for MMS decommissioning, this map identifies a significant body of available primary literature relating to the presence of MMS in the sea, which can be used to indirectly support and underpin decision-making related to the potential biological and ecological effects of their decommissioning, However, it should be noted that most of the evidence is 'post-MMS installation' rather than a true assessment of 'before-after' impact (for instance using a B/A or BACI design), such that the assessment of MMS impact is only correlative (e.g. using a control vs. impact study design or an ‘After-only’ design). Evidence of socio-economic effects was much more limited (7% of articles related to social outcomes, 4% to economic outcomes) compared to that of biological (81%) and ecological effects (48%), a result in keeping with the conclusions of [67]. Given that spatial management, which includes decommissioning of MMS, is a social construct and its success depends on socio-economic factors [68–70], we also highlight that the paucity of evidence related to this field (see [10]) is a significant knowledge gap that places at risk environmental management objectives of MMS decommissioning legislation.

The outputs of this systematic map (i.e., the map database) provides a resource that will help us to (1) improve our understanding of the effects of MMS in the marine environments, as well as (2) identify gaps in knowledge to support research, (3) consider introducing additional strategic funding support to fill these gaps in the future, and (4) compare and contrast the outcomes of primary literature against those of the grey literature to determine if changes in policy are needed.

### Implications for research

This systematic map revealed a significant and disproportionate research investment in improving our understanding of the biological and ecological effects of MMS in the sea. Adversely, considerably less investment has been granted regarding the effects of MMS on physico-chemical, whole habitat, economic, socio-cultural and functional outcome measures, leading to key knowledge gaps. Similarly, there was a disproportionately greater focus on ARs and O&G infrastructure over other MMS exposure types. However, there has been a marked increase in studies focusing on MREIs and OWFs since 2000, reflecting their relatively recent introduction to the environment, coinciding with the global drive toward the use of renewable energy sources. Despite the number of structures that are being, or will require to be, decommissioned in the near future, few studies have assessed the direct effects of their decommissioning on the marine environment, thus a clear knowledge gap still remains with this regard, which should be the focus of urgent primary research.

The lack of evaluation studies addressing these knowledge gaps indicates that there is insufficient evidence to support informed decision-making in many areas and further research is required if an ecosystem-based approach to the decommissioning of MMS is to be achieved. From an ecological standpoint, the paucity of studies describing effects on the physical habitat is perhaps most notable given the increasingly recognised importance of habitat extent and its role in promoting seascape connectivity as a driver of changes in biodiversity and biogeography [70]. From a social and economic perspective, the limited body of evidence related to marine MMS effects on services, may reflect 'out of sight, out of mind' scenarios and attitudes (sensu: plastic waste [71]; offshore fishing [72]; insects [73]) such that investment in research and recognition within policy is negatively correlated with 'observable' effects. Although there is a significant global evidence-base related to MMS that has continued to burgeon over the past two decades, this systematic map suggests that considerable gaps in knowledge remain, in particular with regard to direct evidence for the effects of decommissioning, such that decisions and choices of decommissioning options may deliver sub-optimal ecological and socio-economic outcomes. A greater understanding of the effectiveness of decommissioning options is required.

## Supplementary Information

**Additional file 1. **Authors’ declaration and checklist of adherence to the ROSES guidelines.

**Additional file 2. **List of peer-reviewed literature sources searched, and detail of search strings used.

**Additional file 3. **Lists of articles included, unobtainable or excluded at full text screening with reasons for exclusion.

**Additional file 4. **DREAMS systematic map database.

**Additional file 5. **R code used to create the geographical maps.

**Additional file 6. **Raw data used to create the geographical maps.

**Additional file 7. **Additional table and data.

## Data Availability

All data generated or analysed during this study are included in this published article and its additional files.
