# Correction: Evidence for the effects of decommissioning man-made structures on marine ecosystems globally: a systematic map

**DOI:** 10.1186/s13750-022-00293-9

**Published:** 2022-12-28

**Authors:** Anaëlle J. Lemasson, Paul J. Somerfield, Michaela Schratzberger, Caroline Louise McNeill, Joana Nunes, Christine Pascoe, Stephen C. L. Watson, Murray S. A. Thompson, Elena Couce, Antony M. Knights

**Affiliations:** 1 School of Biological and Marine Sciences, University of Plymouth, Drake Circus, Plymouth, PL4 8AA UK; 2 PML –Plymouth Marine Laboratory, Prospect Place, The Hoe, Plymouth, PL1 3DH UK; 3 Cefas - Centre for Environment, Fisheries and Aquaculture Science, Lowestoft Laboratory, Pakefield Road, Lowestoft, NR33 0HT Suffolk UK

**Correction: Environmental Evidence (2022) 11:35** https://doi.org/10.1186/s13750-022-00285-9

In the original publication of the article [1], the authors identified that the caption of the Additional files 3 and 4 has been swapped.

Correct captions are:

- **Additional file 3.** Lists of articles included, unobtainable or excluded at full text screening with reasons for exclusion.
- **Additional file 4.** DREAMS systematic map database.

The Original article has been corrected.
