# Supplementary material for: Evidence for the effects of decommissioning man-made structures on marine ecosystems globally: a systematic map
Source: Environ Evid. 2022 Nov 1;11:35. doi: 10.1186/s13750-022-00285-9 (PMC11378805; doi:10.1186/s13750-022-00285-9)
Supplement: Supplementary file 7 — Additional file 7. Additional table and data. [file 13750_2022_285_MOESM7_ESM.docx]

**Additional file 6: Supplementary table**


**Table S1 Distribution of articles by MMS type across and within oceanic regions.**

| **MMS type within oceanic region** | **Count of unique articles** | **% of total unique articles** | **% of unique articles within the oceanic region*** |
| --- | --- | --- | --- |
| **Arctic Ocean** | **5** | **0.51%** |  |
| Oil and gas | 4 | 0.41% | 80.00% |
| Shipwreck | 1 | 0.10% | 20.00% |
| Artificial reef | 0 | 0% | 0% |
| MREI | 0 | 0% | 0% |
| Offshore wind farm | 0 | 0% | 0% |
| Other similar MMS | 0 | 0% | 0% |
| Unspecified cables or pipelines | 0 | 0% | 0% |
| **equatorial Atlantic** | **5** | **0.51%** |  |
| Oil and gas | 45 | 0.51% | 100.00% |
| Artificial reef | 0 | 0% | 0% |
| MREI | 0 | 0% | 0% |
| Offshore wind farm | 0 | 0% | 0% |
| Other similar MMS | 0 | 0% | 0% |
| Shipwreck | 0 | 0% | 0% |
| Unspecified cables or pipelines | 0 | 0% | 0% |
| **equatorial Pacific** | **8** | **0.82%** |  |
| Shipwreck | 6 | 0.61% | 75.00% |
| Artificial reef | 2 | 0.20% | 25.00% |
| MREI | 0 | 0% | 0% |
| Offshore wind farm | 0 | 0% | 0% |
| Oil and gas | 0 | 0% | 0% |
| Other similar MMS | 0 | 0% | 0% |
| Unspecified cables or pipelines | 0 | 0% | 0% |
| **Global** | **12** | **1.23%** |  |
| Artificial reef | 6 | 0.61% | 50.00% |
| Oil and gas | 6 | 0.61% | 50.00% |
| Offshore wind farm | 3 | 0.31% | 25.00% |
| Shipwreck | 2 | 0.20% | 16.67% |
| MREI | 1 | 0.10% | 8.33% |
| Unspecified cables or pipelines | 1 | 0.10% | 8.33% |
| Other similar MMS | 0 | 0% | 0% |
| **Gulf of Mexico** | **124** | **12.67%** |  |
| Artificial reef | 63 | 6.44% | 50.81% |
| Oil and gas | 61 | 6.23% | 49.19% |
| Shipwreck | 25 | 2.55% | 20.16% |
| Offshore wind farm | 1 | 0.10% | 0.81% |
| MREI | 0 | 0% | 0% |
| Other similar MMS | 0 | 0% | 0% |
| Unspecified cables or pipelines | 0 | 0% | 0% |
| **Indian Ocean** | **66** | **6.74%** |  |
| Artificial reef | 43 | 4.39% | 65.15% |
| Oil and gas | 18 | 1.84% | 27.27% |
| Shipwreck | 8 | 0.82% | 12.12% |
| MREI | 1 | 0.10% | 1.52% |
| Offshore wind farm | 1 | 0.10% | 1.52% |
| Other similar MMS | 0 | 0% | 0% |
| Unspecified cables or pipelines | 0 | 0% | 0% |
| **Mediterranean Sea** | **119** | **12.16%** |  |
| Artificial reef | 79 | 8.07% | 66.39% |
| Oil and gas | 22 | 2.25% | 18.49% |
| Shipwreck | 19 | 1.94% | 15.97% |
| Offshore wind farm | 2 | 0.20% | 1.68% |
| Other similar MMS | 1 | 0.10% | 0.84% |
| MREI | 0 | 0% | 0% |
| Unspecified cables or pipelines | 0 | 0% | 0% |
| **North Sea** | **122** | **12.46%** |  |
| Offshore wind farm | 67 | 6.84% | 54.92% |
| Oil and gas | 38 | 3.88% | 31.15% |
| Shipwreck | 17 | 1.74% | 13.93% |
| MREI | 8 | 0.84% | 6.56% |
| Other similar MMS | 7 | 0.72% | 5.74% |
| Artificial reef | 4 | 0.41% | 3.28% |
| Unspecified cables or pipelines | 2 | 0.20% | 1.64% |
| **north-east Atlantic** | **142** | **14.50%** |  |
| Artificial reef | 53 | 5.41% | 37.32% |
| Offshore wind farm | 40 | 4.09% | 28.17% |
| MREI | 33 | 3.37% | 23.24% |
| Shipwreck | 8 | 0.82% | 5.63% |
| Other similar MMS | 8 | 0.82% | 5.63% |
| Oil and gas | 6 | 0.61% | 4.23% |
| Unspecified cables or pipelines | 0 | 0% | 0% |
| **north-east Pacific** | **91** | **9.30%** |  |
| Artificial reef | 46 | 4.70% | 50.55% |
| Oil and gas | 41 | 4.19% | 45.05% |
| Shipwreck | 6 | 0.61% | 6.59% |
| MREI | 1 | 0.10% | 1.10% |
| Offshore wind farm | 1 | 0.10% | 1.10% |
| Other similar MMS | 0 | 0% | 0% |
| Unspecified cables or pipelines | 0 | 0% | 0% |
| **north-west Atlantic** | **122** | **12.46%** |  |
| Artificial reef | 81 | 8.27% | 66.39% |
| Shipwreck | 25 | 2.55% | 20.49% |
| Offshore wind farm | 14 | 1.43% | 11.48% |
| Oil and gas | 7 | 0.72% | 5.74% |
| MREI | 3 | 0.31% | 2.46% |
| Other similar MMS | 2 | 0.20% | 1.64% |
| Unspecified cables or pipelines | 0 | 0% | 0% |
| **north-west Pacific** | **81** | **8.27%** |  |
| Artificial reef | 69 | 7.05% | 85.19% |
| Shipwreck | 8 | 0.82% | 9.88% |
| Oil and gas | 4 | 0.41% | 4.94% |
| Offshore wind farm | 3 | 0.31% | 3.70% |
| MREI | 1 | 0.10% | 1.23% |
| Other similar MMS | 1 | 0.10% | 1.23% |
| Unspecified cables or pipelines | 0 | 0% | 0% |
| **south-east Atlantic** | **3** | **0.31%** |  |
| Artificial reef | 2 | 0.20% | 66.67% |
| Oil and gas | 1 | 0.10% | 33.33% |
| MREI | 0 | 0% | 0% |
| Offshore wind farm | 0 | 0% | 0% |
| Other similar MMS | 0 | 0% | 0% |
| Shipwreck | 0 | 0% | 0% |
| Unspecified cables or pipelines | 0 | 0% | 0% |
| **south-east Pacific** | **5** | **0.51%** |  |
| Artificial reef | 4 | 0.41% | 80.00% |
| Oil and gas | 1 | 0.10% | 20.00% |
| Shipwreck | 0 | 0% | 0% |
| MREI | 0 | 0% | 0% |
| Offshore wind farm | 0 | 0% | 0% |
| Other similar MMS | 0 | 0% | 0% |
| Unspecified cables or pipelines | 0 | 0% | 0% |
| **south-west Atlantic** | **43** | **4.39%** |  |
| Artificial reef | 28 | 2.86% | 65.12% |
| Shipwreck | 14 | 1.43% | 32.56% |
| Oil and gas | 3 | 0.31% | 6.98% |
| MREI | 0 | 0% | 0% |
| Offshore wind farm | 0 | 0% | 0% |
| Other similar MMS | 0 | 0% | 0% |
| Unspecified cables or pipelines | 0 | 0% | 0% |
| **south-west Pacific** | **38** | **3.88%** |  |
| Artificial reef | 28 | 2.86% | 73.68% |
| Shipwreck | 9 | 0.92% | 23.68% |
| Offshore wind farm | 1 | 0.10% | 2.63% |
| Other similar MMS | 1 | 0.10% | 2.63% |
| MREI | 0 | 0% | 0% |
| Oil and gas | 0 | 0% | 0% |
| Unspecified cables or pipelines | 0 | 0% | 0% |

*Grand total may be greater than the number of unique articles (N=979), and percentages may add up to more than 100%, as some articles contains studies spanning multiple MMS types.
